# *APOE4* is Associated with Differential Regional Vulnerability to Bioenergetic Deficits in Aged *APOE* Mice

**DOI:** 10.1038/s41598-020-61142-8

**Published:** 2020-03-09

**Authors:** Estela Area-Gomez, Delfina Larrea, Marta Pera, Rishi R. Agrawal, David N. Guilfoyle, Leila Pirhaji, Kathleen Shannon, Hirra A. Arain, Archana Ashok, Qiuying Chen, Allissa A. Dillman, Helen Y. Figueroa, Mark R. Cookson, Steven S. Gross, Ernest Fraenkel, Karen E. Duff, Tal Nuriel

**Affiliations:** 1 Department of Neurology, Columbia University, 630 West 168th Street, New York, NY 10032 USA; 2 Institute of Human Nutrition, Columbia University, 630 West 168th Street, New York, NY 10032 USA; 3 Department of Basic Sciences, Facultat de Medicina i Ciències de la Salut, Universitat Internacional de Catalunya (UIC), 08195 Sant Cugat del Vallés, Spain; 4 Center for Biomedical Imaging and Neuromodulation, Nathan S. Kline Institute, Orangeburg, NY 10962 USA; 5 Department of Biological Engineering, Massachusetts Institute of Technology, 350 Brookline Street, Cambridge, MA 02139 USA; 6 Animal Facility, Nathan S. Kline Institute, Orangeburg, NY 10962 USA; 7 Taub Institute for Research on Alzheimer’s Disease and the Aging Brain, Columbia University, 630 West 168th Street, New York, NY 10032 USA; 8 Department of Pathology and Cell Biology, Columbia University, 630 West 168th Street, New York, NY 10032 USA; 9 Department of Pharmacology, Weill Cornell Medical College, 1300 York Avenue, New York, NY 10065 USA; 10 Cell Biology and Gene Expression Section, Laboratory of Neurogenetics, National Institute on Aging, National Institutes of Health, Bethesda, Maryland 20892 USA; 11 Broad Institute, 415 Main Street, Cambridge, MA 02142, Cambridge, Massachusetts, 02139 USA; 12 UK Dementia Research Institute, University College London, Cruciform Building, Gower Street, London, WC1E 6BT UK

**Keywords:** Alzheimer's disease, Molecular neuroscience

## Abstract

The ε4 allele of apolipoprotein E (*APOE*) is the dominant genetic risk factor for late-onset Alzheimer’s disease (AD). However, the reason for the association between *APOE4* and AD remains unclear. While much of the research has focused on the ability of the apoE4 protein to increase the aggregation and decrease the clearance of Aβ, there is also an abundance of data showing that *APOE4* negatively impacts many additional processes in the brain, including bioenergetics. In order to gain a more comprehensive understanding of *APOE4*′s role in AD pathogenesis, we performed a transcriptomics analysis of *APOE4* vs. *APOE3* expression in the entorhinal cortex (EC) and primary visual cortex (PVC) of aged *APOE* mice. This study revealed EC-specific upregulation of genes related to oxidative phosphorylation (OxPhos). Follow-up analysis utilizing the Seahorse platform showed decreased mitochondrial respiration with age in the hippocampus and cortex of *APOE4* vs. *APOE3* mice, but not in the EC of these mice. Additional studies, as well as the original transcriptomics data, suggest that multiple bioenergetic pathways are differentially regulated by *APOE4* expression in the EC of aged *APOE* mice in order to increase the mitochondrial coupling efficiency in this region. Given the importance of the EC as one of the first regions to be affected by AD pathology in humans, the observation that the EC is susceptible to differential bioenergetic regulation in response to a metabolic stressor such as *APOE4* may point to a causative factor in the pathogenesis of AD.

## Introduction

Possession of the ε4 allele of apolipoprotein E (*APOE*) is the major genetic risk factor for late-onset Alzheimer’s disease (AD). In normal physiology, the apoE protein plays a vital role in the transport of cholesterol and other lipids through the bloodstream, as well as within the brain^1–3^. Although the three common isoforms of apoE (E2, E3 and E4) differ from each other at only two amino acids—apoE2 (Cys112, Cys158), apoE3 (Cys112, Arg158), apoE4 (Arg112, Arg158)—this small change in amino acid sequence has a large effect on the protein structure, resulting in differential affinities towards apoE’s lipid cargo, as well as its receptors [see reviews by^1,4^].

Importantly, these isoform differences also have a major impact on the pathogenesis of late-onset AD. Although the *APOE2, APOE3* and *APOE4* alleles are normally present at a relative frequency of about 8%, 78% and 14%, respectively, the *APOE4* allele is present at a relative frequency of about 37% in AD patients^5^, with individuals who possess one or two *APOE4* alleles having an odds ratio for AD of about 3 or 12, respectively^5,6^. While a number of mechanisms have been proposed to help explain this *APOE4*-associated susceptibility to AD, the precise cause remains a source of debate. One prominent hypothesis is that this increase in AD risk is due to the capability of apoE4 to increase the aggregation and decrease the clearance of Aβ^7–13^. However, *APOE4* expression has also been shown to have deleterious effects on numerous Aβ-independent pathways, including lipid metabolism, tau pathology, bioenergetics, neuronal development, synaptic plasticity, the neuro-vasculature, and neuro-inflammation [see reviews by^14^ and^15^], any number of which could play an important role in the pathogenesis of AD among *APOE4* carriers.

In terms of *APOE4*′s effects on bioenergetics, a number of pivotal reports have demonstrated that *APOE4* expression leads to widespread dysregulation of the brain’s bioenergetic capacity. For example, early reports by Reiman and colleagues demonstrated that both young and old *APOE4* carriers display decreased glucose utilization, as measured by fluorodeoxyglucose positron emission tomography (FDG-PET), in brain regions similar to those seen with AD patients^16–18^. Additional reports have detailed the wide range of bioenergetic insults that *APOE4* expression can cause in the brain, including impaired insulin signaling^19,20^, reduced cerebral blood volume and cognitive function in response to a high fat diet (HFD)^21,22^, altered genetic expression of glucose-regulating enzymes and transporters^23,24^, and the generation of a toxic C-terminal fragment of apoE4 that can directly target electron transport chain (ETC) complexes in the mitochondria^25–27^.

In order to study the diverse effects that *APOE4* expression has on the brain, we performed a transcriptomics analysis on the entorhinal cortex (EC) and primary visual cortex (PVC) of 14–15 month-old *APOE* targeted replacement mice, which express human *APOE* in place of their mouse *Apoe* gene and which do not develop overt AD pathology^28,29^. In addition to other observations^30^, this transcriptomics analysis revealed the differential regulation of numerous genes related to energy metabolism. Follow up studies showed that, while aged *APOE4* mice possess bioenergetic deficits in the hippocampus (Hip) and cortex (Ctx), the EC of these mice appears to possess unique counterbalancing mechanisms that allow it to resist these *APOE4*-associated decreases in mitochondrial respiration. We hypothesize that this differential regulation of EC bioenergetics may play an important role in the pathogenesis of AD.

## Results

### Transcriptomics analysis reveals differential expression of energy-related genes in the EC of aged *APOE4* mice

**Figure 1:**
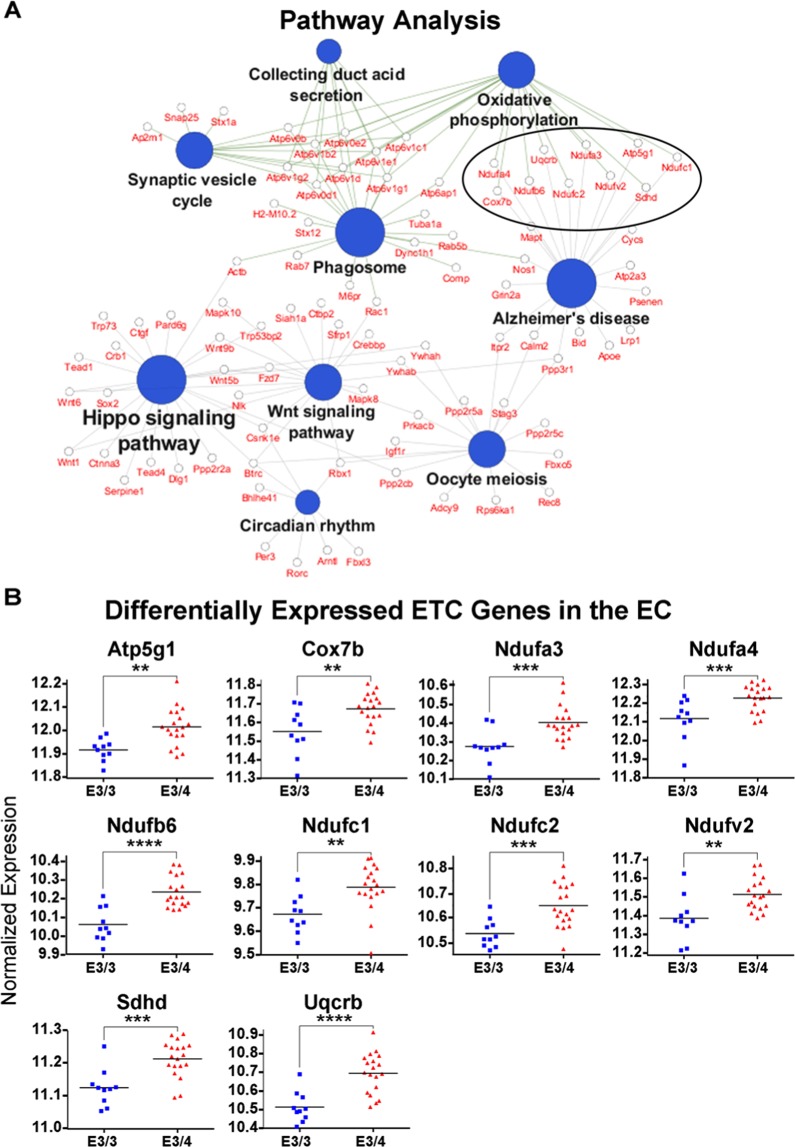
Transcriptomics analysis reveals an upregulation of electron transport chain genes in the EC of aged male *APOE4* mice. RNA-sequencing was performed in order to analyze the effects of differential *APOE* isoform expression on RNA levels in the EC and PVC of aged *APOE* mice (19 *APOE3/4* males vs. 10 *APOE3/3* males). (**A**) Shown here are the significantly enriched KEGG pathways observed in the EC of *APOE3/4* vs. *APOE3/3* mice, using Cytoscape’s ClueGo application, with the ten differentially expressed electron transport chain (ETC) genes circled. (**B**) Graphs of the differentially regulated ETC genes in the EC of the *APOE3/4* vs. *APOE3/3* mice. (**denotes p < 0.01; ***denotes *p* < 0.001; ****denotes p < 0.0001).

In order to investigate the effects of *APOE4* expression in an untargeted manner, we performed a transcriptomics analysis on RNA extracted from a brain region that is acutely vulnerable to AD pathology (the EC) vs. a less vulnerable brain region (the PVC) of 14–15 month-old *APOE* targeted replacement mice (10 *APOE3/3* and 19 *APOE3/4* males). The raw data generated from this analysis (Supplementary Tables S1 and S2) has already been published as part of a separate study on *APOE4*′s impact on endosomal-lysosomal dysregulation^30^. However, as is often the case with omics studies, the large amount of data generated from the analysis can inform us about changes in multiple biological pathways that cannot all be investigated in a single study. In this case, in addition to the dysregulation of endosomal-lysosomal genes that were revealed in our pathway analysis, another major KEGG pathway that was observed to be significantly enriched for differentially expressed genes was “Oxidative Phosphorylation” (Fig. 1A). This enrichment was driven by a large number of differentially expressed genes encoding for subunits of ETC complexes I-V, with each of these genes upregulated in the EC of aged male *APOE3/4* mice, as compared to aged male *APOE3/3* mice (Fig. 1B).

### Seahorse analysis reveals decreased mitochondrial respiration with aging in the hippocampus and cortex of *APOE4* vs. *APOE3* mice, but not in the EC

**Figure 2:**
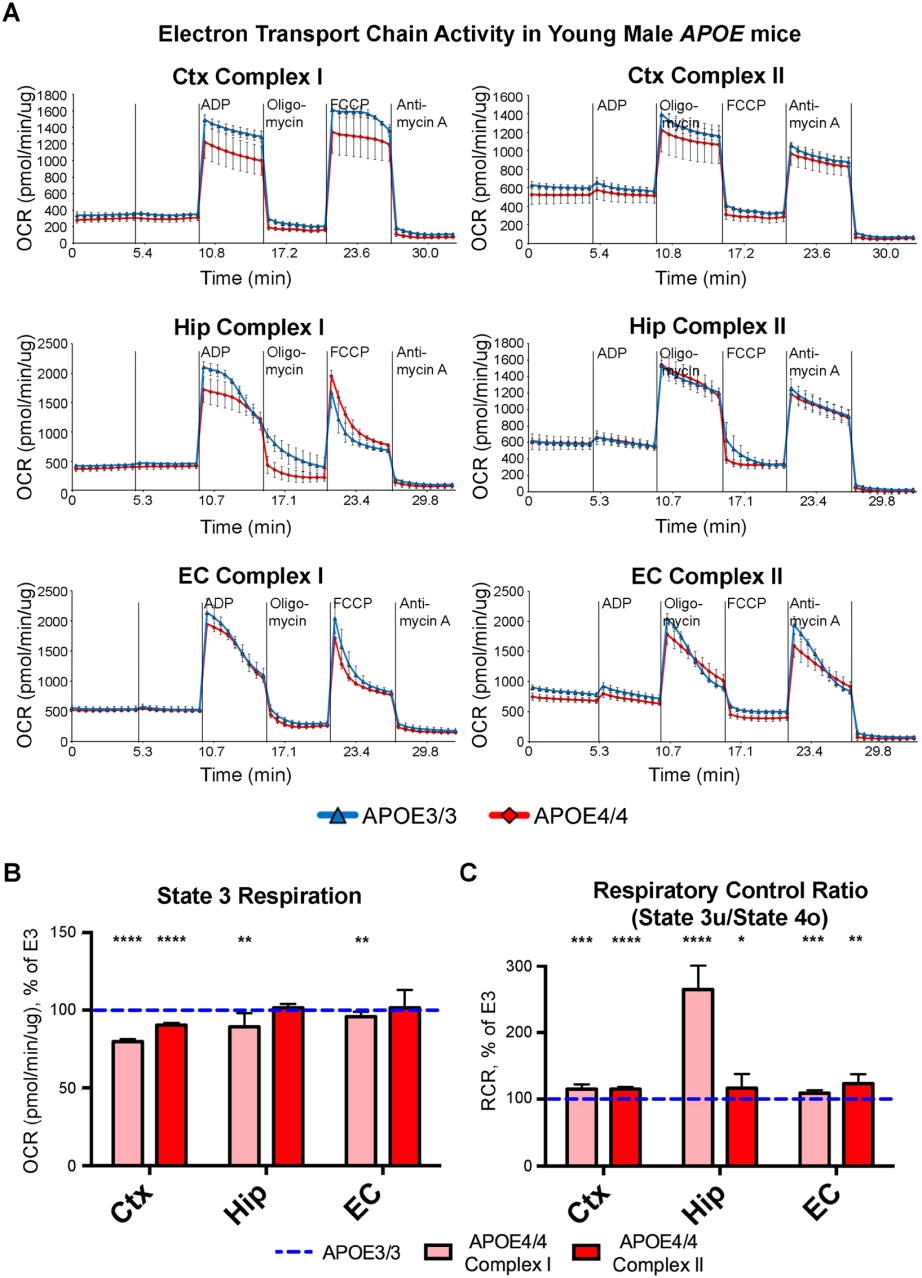
Seahorse analysis reveals minimal differences in mitochondrial respiration in the cortex, hippocampus, and EC of young male *APOE4* mice. Seahorse analysis was performed in order to analyze the effects of differential *APOE* isoform expression on mitochondrial respiration in mitochondria that were isolated from the cortex (Ctx), hippocampus (Hip) and EC of 6-month-old *APOE* mice (3 *APOE4/4* males, tissues pooled vs. 3 *APOE3/3* males, tissues pooled). (**A**) The oxygen consumption rate (OCR) from each region shows minor reductions in Complex I-mediated mitochondrial respiration in the Ctx, but not in Complex II-mediated mitochondrial respiration in the Ctx or in Complex I- or Complex II-mediated mitochondrial respiration in the Hip and EC of the young *APOE4/4* mice. (**B**,**C**) Bar graphs showing the average OCR from (**B**) State 3 and for (**C**) the Respiration Control Ratio (RCR; state 3 u/state 4o) in each region of the *APOE4/4* mice, as a percentage of the *APOE3/3* OCR from the equivalent tissues. The dotted blue line represents the normalized levels in the *APOE3/3* tissues. (*denotes p < 0.05; **denotes p < 0.01; ***denotes *p* < 0.001; ****denotes p < 0.0001).

**Figure 3:**
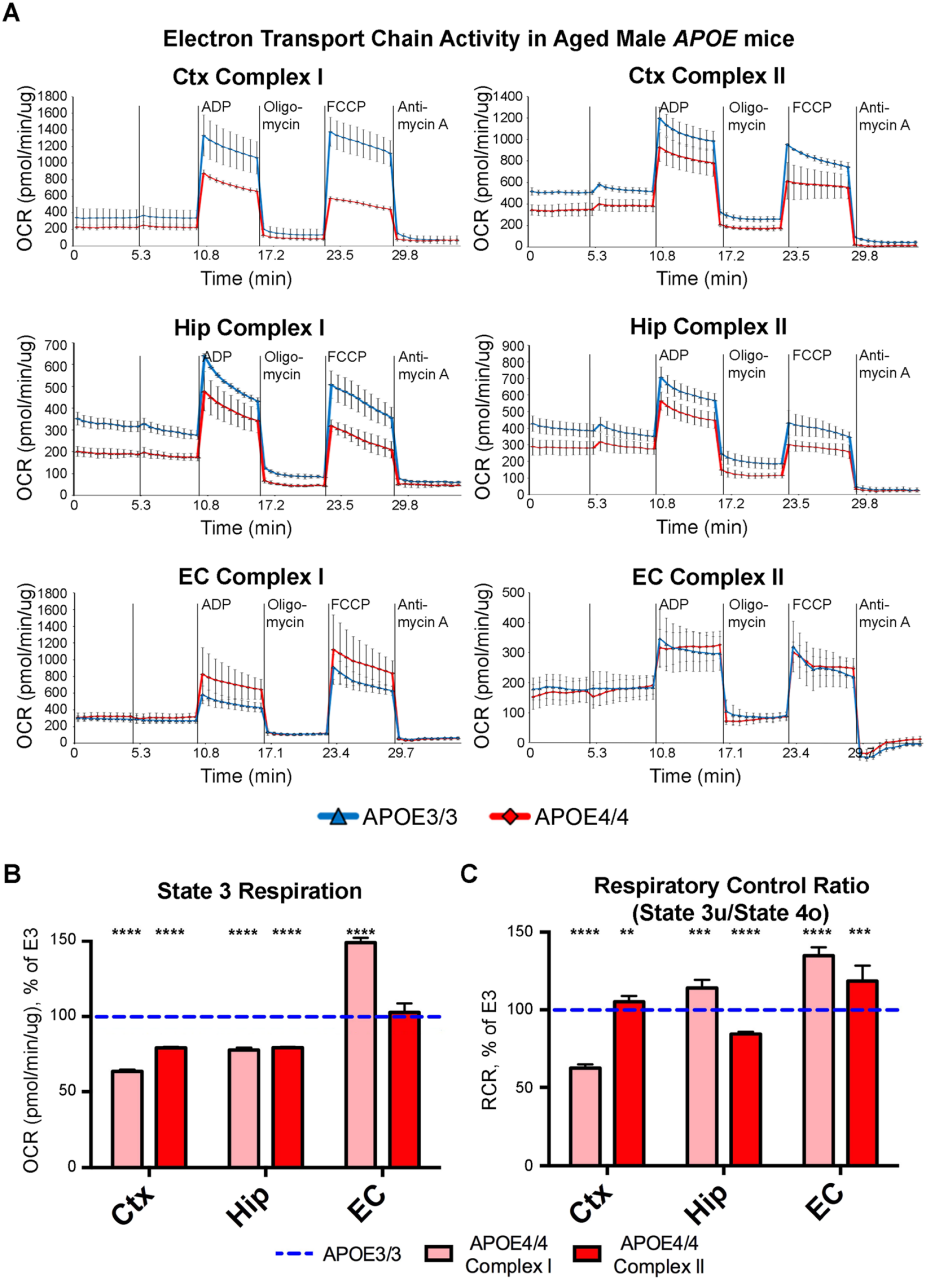
Seahorse analysis reveals decreased mitochondrial respiration in the cortex and hippocampus, but not in the EC, of aged male *APOE4* mice. Seahorse analysis was performed in order to analyze the effects of differential *APOE* isoform expression on mitochondrial respiration in mitochondria that were isolated from the cortex (Ctx), hippocampus (Hip) and EC of 20-month-old *APOE* mice (2 *APOE4/4* males, tissues pooled vs. 2 *APOE3/3* males, tissues pooled). (**A**) The oxygen consumption rate (OCR) from each region shows decreased mitochondrial respiration in the Ctx and Hip, but not the EC of the aged *APOE4/4* mice. (**B**,**C**) Bar graphs showing the average OCR from (**B**) State 3 and for (**C**) the Respiration Control Ratio (RCR; state 3 u/state 4o) in each region of the *APOE4/4* mice, as a percentage of the *APOE3/3* OCR from the equivalent tissues. The dotted blue line represents the normalized levels in the *APOE3/3* tissues. (**denotes p < 0.01; ***denotes *p* < 0.001; ****denotes p < 0.0001).

In order to validate and expand upon the differential expression of oxidative phosphorylation (OxPhos) genes that we observed in our transcriptomics analysis, we conducted a series of Seahorse experiments on mitochondria isolated from the EC and other important brain regions of *APOE4/4* vs. *APOE3/3* mice. This *APOE4/4* vs. *APOE3/3* comparison was utilized in order to maximize the *APOE* genotype-associated differences observed with our original transcriptomics analysis on *APOE3/4* vs. *APOE3/3* mice. Utilizing the Seahorse XF24 platform, we first measured the oxygen consumption rate (OCR) as a measure of mitochondrial respiration from the EC and PVC of 15-month-old *APOE* mice (4 *APOE3/3* and 4 *APOE4/4* males; samples pooled within each genotype and region). As shown in Supplementary Fig. S1, we observed a significant reduction in both the Complex I-mediated and Complex II-mediated respiration in mitochondria from the PVC of these *APOE4/4* vs. *APOE3/3* mice, but no difference in the respiration between genotypes in the mitochondria from the EC region. In order to investigate whether this decreased mitochondrial respiration in the PVC was unique to the PVC, or whether it represented a more global decrease in mitochondrial function within the aged *APOE4* brain, we performed additional experiments measuring the OCR of mitochondria isolated from the EC, Hip and Ctx of 6-month-old *APOE* mice (3 *APOE3/3* and 3 *APOE4/4* males; samples pooled within each genotype and region) and 20-month-old *APOE* mice (2 *APOE3/3* and 2 *APOE4/4* males; samples pooled within each genotype and region). As shown in Fig. 2, in the presence of pyruvate-malate (Complex I-mediated activity), we observed a reduction in State 3 respiration of mitochondria from the Ctx of 6-month-old male *APOE4/4* vs. *APOE3/3* mice, and very slight reductions in State 3 respiration of mitochnodria from the Hip and EC of these mice. On the other hand, as shown in Fig. 3, there were significant reductions in Complex I- and Complex II-mediated respiration of mitochondria from both the Hip and the Ctx of 20-month-old male *APOE4/4* vs. *APOE3/3* mice. Intriguingly, however, in mitochondria from the EC of these 20-month-old *APOE4/4* vs. *APOE3/3* mice, we did not observe any significant differences in basal mitochondrial respiration in Complex I- or Complex II-mediated respiration, and we actually observed significant increases in State 3 respiration in Complex I-mediated respiration in mitochondria from the EC (Fig. 3B). Furthermore, using the data generated from this Seahorse analysis, we determined the respiratory control ratio (RCR) (state3u/state4o), which is a measure of the coupling efficiency between OxPhos and ATP production^31,32^ (Fig. 3C). This analysis indicates that EC mitochondria from the 20-month-old *APOE4/4* mice are highly coupled, resulting in increased production of ATP per unit of oxygen.

These observations suggest that, while *APOE4* expression leads to an overall reduction in mitochondrial function in the brains of aged *APOE4* mice, which may be related to other *APOE4*-associated bioenergetic deficits observed using alternative approaches^19–24^, mitochondria in the EC seem to undergo differential bioenergetic regulation, characterized by increased coupling efficiency. Importantly, these region-specific differences in the effects of *APOE4* expression on mitochondrial respiration did not appear to be the result of changes in mitochondrial mass, as the levels of Tom20 protein, as well as the levels of PGC1α RNA and the ratio of Mitochondrial:Nuclear DNA, were unchanged between *APOE* genotypes in the EC, Hip, and Ctx of 21-month-old male *APOE* mice (Supplementary Fig. S2A,C,D). Differential *APOE* isoform expression also did not result in changes in the levels of specific ETC complex proteins in the EC, Hip or Ctx (Supplementary Fig. S2B).

In order to investigate whether there is a gender-effect associated with these region-specific differences in bioenergetic regulation, we also measured the OCR of mitochondria isolated from the EC, Hip and Ctx of 20-month-old female *APOE* mice (2 *APOE3/3* and 2 *APOE4/4* females; samples pooled within each genotype and region). As shown in Supplementary Fig. S3, the aged female *APOE4/4* mice also showed a reduction in State 3 respiration in Complex I-mediated respiration in mitochondria from the Ctx, but not the EC. However, the Complex I-mediated respiration deficits in the Ctx were not nearly as robust in the female mice as they were in the males. Interestingly, in mitochondria from both the Ctx and the EC of these aged female *APOE4/4* mice, we also observed a significant increase in Complex II-mediated (but not Complex I-mediated) State 3 respiration, suggesting that increased Complex II-mediated mitochondrial respiration (most likely from increased ß-oxidation of fatty acids) might prevent these aged female *APOE4/4* mice from developing the levels of reduced Complex I-mediated mitochondrial respiration observed in the aged male *APOE4/4* mice.

### Computational analysis of untargeted metabolomics data reveals differential expression of ketones in the EC of aged *APOE4* mice

**Figure 4:**
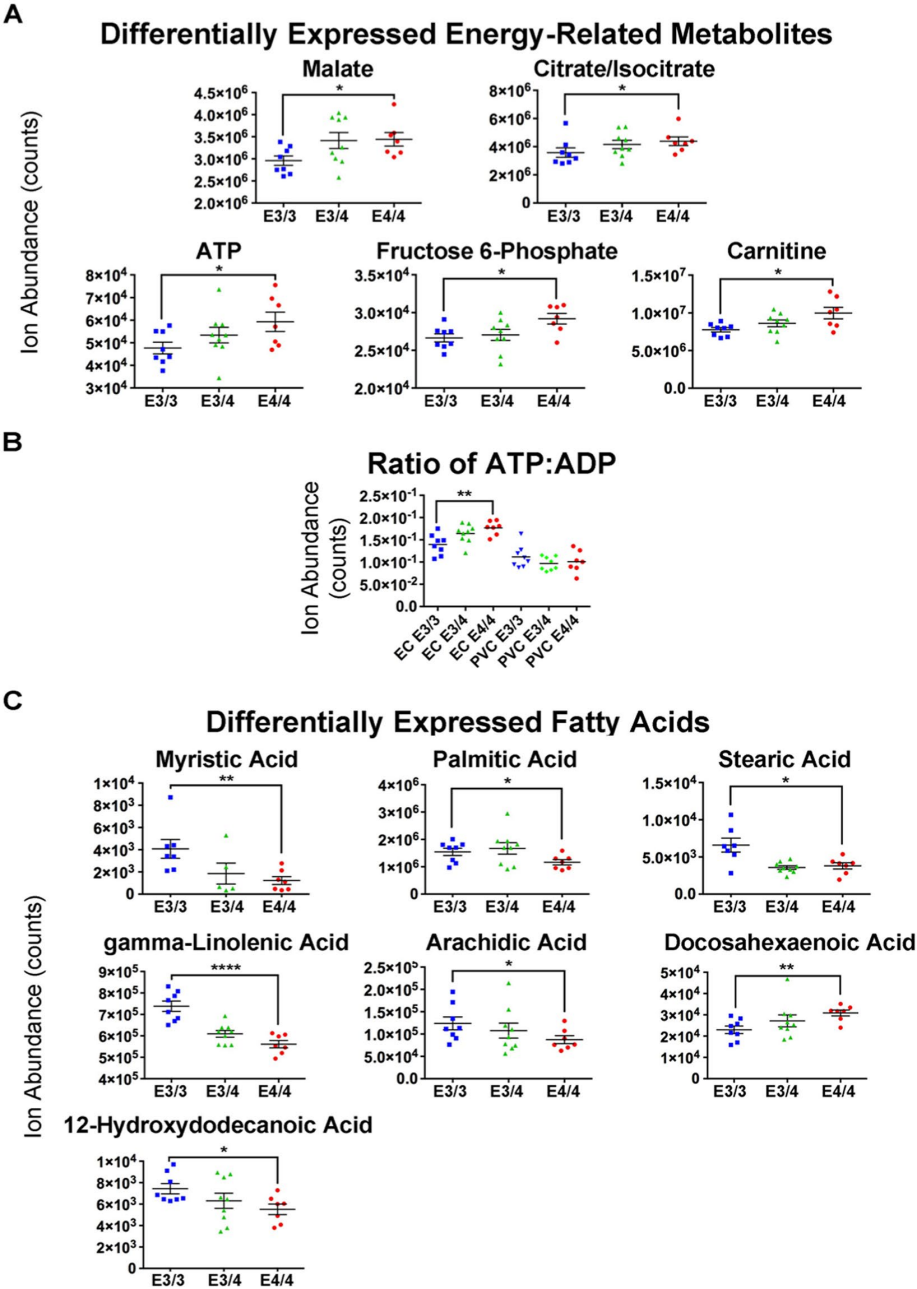
Metabolomics analysis reveals differential levels of energy-related metabolites and fatty acids in the EC of aged male *APOE4* mice. An untargeted metabolomics analysis was performed in order to analyze the effects of differential *APOE* isoform expression on metabolite levels in the EC and PVC of aged *APOE* mice (8 *APOE3/3*, 9 *APOE3/4* and 7 *APOE4/4* males). (**A**–**C**) Shown here are graphs of the bioenergetics-related metabolites that were shown to be differentially expressed in the EC of the *APOE4/4* vs. *APOE3/3* mice: (**A**) general energy-related metabolites, (**B**) the ratio of ATP:ADP in each genotype group from the EC and PVC of the aged *APOE* mice, and (C) fatty acids. (*denotes p < 0.05; **denotes p < 0.01; ****denotes p < 0.0001).

In order to investigate the source of these differential bioenergetic effects in the EC, we conducted a series of additional analyses on the EC of aged male *APOE4/4* vs *APOE3/3* mice. We have already published on an untargeted metabolomics study that we performed on metabolites extracted from the EC and PVC of 14–15 month-old male *APOE4/4* vs. *APOE3/3* mice, which is part of a larger study elucidating the effects *APOE4* expression on neuronal hyperactivity^33^. Intriguingly, this metabolomics study uncovered increases in numerous energy-related metabolites, including several TCA cycle metabolites (malate, citrate and isocitrate), as well as fructose-6-phosphate, carnitine, and ATP, each of which had higher levels in the EC of the aged *APOE4/4* vs. *APOE3/3* mice (Fig. 4A). Furthermore, the metabolomics data indicates a higher ATP:ADP ratio in the EC of these aged *APOE4/4* vs. *APOE3/3* mice (Fig. 4B), which, in light of our transcriptomics data, suggests an increase in the rate of oxidative metabolism in this region. Furthermore, our metabolomics data also revealed an *APOE4*-associated decrease in the levels of numerous free fatty acids in aged male *APOE4/4* vs. *APOE3/3* mice (Fig. 4C), which, unlike each of the previously mentioned energy-related metabolites (with the exception of carnitine), were differentially altered in both the EC and the PVC. These changes in fatty acid and carnitine levels are suggestive of differences in β-oxidation activity in both the EC and the PVC of these aged male *APOE4* mice.

In addition to these and other differentially expressed metabolites that were identified during the course of this untargeted metabolomics study (Tables 1 and 2), there were also hundreds of additional differentially expressed metabolites that we were unable to identify using the tools that were available to us at the time that this study was conducted. Therefore, in order to extract more information out of this untargeted metabolomics dataset, we utilized the PIUMet (Prize-collecting Steiner forest algorithm for Integrative Analysis of Untargeted Metabolomics) method^34^ to predict the identities of these differentially expressed untargeted metabolites from the EC of aged *APOE4/4* vs. *APOE3/3* mice. PIUMet uses network optimization to analyze the data in the context of a vast database of protein-protein and protein-metabolite interactions, revealing both known and uncharacterized pathways that contain the putative metabolites.

**Table 1:** Differentially expressed targeted metabolites from the EC of aged male *APOE4/4* vs. *APOE3/3* mice.

Metabolite	Regulation in E4/4	Fold Change	p-value	FDR	Detection Mode	CAS Number
**Fatty Acids**						
gamma-Linolenic Acid	down	1.31	1.19E-03	0.093	positive	506-26-3
Myristic Acid	down	3.92	0.004	0.118	positive	544-63-8
Docosahexaenoic Acid	up	1.36	0.011	0.154	positive	6217-54-5
Stearic Acid	down	1.69	0.011	0.154	positive	57-11-4
12-Hydroxydodecanoic Acid	down	1.36	0.021	0.213	positive	505-95-3
Arachidic Acid	down	1.40	0.037	0.230	negative	506-30-9
Palmitic Acid	down	1.32	0.049	0.243	negative	57-10-3
**Oligosaccharides**						
trisaccharide	up	1.99	0.001	0.049	negative	512-69-6
tetrasaccharide	up	2.24	0.015	0.169	negative	10094-58-3
disaccharide	up	2.20	0.015	0.169	negative	63-42-3
**Vitamin and Vitamin Derivatives**						
Phylloquinone	up	1.47	0.003	0.102	positive	84-80-0
Tocopherol	up	2.74	0.005	0.123	negative	59-02-9
Dehydroascorbic acid	up	1.76	0.008	0.134	positive	490-83-5
**Energy-Related Metabolites**						
Inosine 5′-monophosphate (IMP)	up	2.26	0.003	0.0720	negative	131-99-7
D-Fructose 6-phosphate	up	1.10	0.011	0.169	negative	643-13-0
Succinoadenosine	up	1.47	0.011	0.169	negative	4542-23-8
Carnitine	up	1.27	0.021	0.213	positive	541-15-1
Citric Acid/Isocitric Acid	up	1.24	0.028	0.230	negative	77-92-9/1637-73-6
Malic Acid	up	1.16	0.037	0.230	negative	97-67-6
ATP	up	1.24	0.049	0.243	negative	56-65-5
**Cholesterol Metabolites**						
Lanosterol	up	2.27	0.015	0.169	negative	79-63-0
Cholesteryl Acetate	up	2.29	0.015	0.169	negative	604-35-3
**Amino Acids**						
Leucine	up	1.26	0.015	0.169	negative	61-90-5
Proline	up	1.12	0.037	0.230	negative	147-85-3
Glycine	up	1.10	0.037	0.230	negative	56-40-6
**Tryptophan Metabolites**						
Quinaldic Acid	up	1.68	0.001	0.049	negative	93-10-7
Kynurenine	up	2.49	0.015	0.169	negative	2922-83-0
Kynurenic Acid	up	1.32	0.049	0.243	negative	492-27-3
**Cysteine and Methionine Metabolites**						
S-Adenosylhomocysteine	up	1.15	0.021	0.213	positive	979-92-0
**Histidine Metabolism**						
Carnosine	up	1.31	0.037	0.230	negative	305-84-0
**Arginine and Proline Metabolites**						
4-Oxoproline	up	1.30	0.003	0.102	positive	4347-18-6
**Tyrosine Metabolites**						
Vanylglycol (MHPG)	up	1.64	0.011	0.169	negative	67423-45-4
Tyramine	up	1.06	0.028	0.216	positive	51-67-2
Thymidine	down	1.49	0.028	0.216	positive	50-89-5
Uracil	down	1.38	0.049	0.243	negative	66-22-8
**Purine Metabolism**						
GMP	up	1.14	0.028	0.230	negative	85-32-5
**Miscellaneous**						
Hydroxybutyric acid	up	1.35	0.002	0.055	negative	5094-24-6
Methylglutarylcarnitine	up	2.42	0.005	0.134	positive	102673-95-0
Trimethylamine N-oxide	up	2.65	0.021	0.213	positive	1184-78-7
2-Hydroxypyridine	up	1.36	0.037	0.275	positive	142-08-5
N-Acetylneuraminic Acid	up	1.23	0.037	0.230	negative	131-48-6

**Table 2:** Differentially expressed targeted metabolites from the PVC of aged male *APOE4/4* vs. *APOE3/3* mice.

Metabolite	Regulation in E4/4	Fold Change	p-value	FDR	Detection Mode	CAS Number
**Fatty Acids**						
gamma-Linolenic Acid	down	1.19	0.003	0.068	positive	506-26-3
Palmitic Acid	down	1.32	0.021	0.212	negative	57-10-3
10-Hydroxydecanoate	up	2.22	0.015	0.195	positive	1679-53-4
Docosahexaenoic Acid	down	2.03	0.021	0.212	negative	6217-54-5
Arachidonic acid	down	1.45	0.021	0.212	negative	506-32-1
**Oligosaccharides**						
trisaccharide	up	1.82	0.015	0.212	negative	
tetrasaccharide	up	2.70	0.021	0.212	negative	
**Vitamin and Vitamin Derivatives**						
Dehydroascorbic acid	up	2.03	0.001	0.062	positive	490-83-5
Phylloquinone	up	1.90	0.001	0.062	positive	84-80-0
Ascorbic acid	up	105.84	0.005	0.192	negative	50-81-7
Tocopherol	up	2.28	0.008	0.192	negative	59-02-9
**Energy-Related Metabolites**						
Acetylcarnitine	up	1.40	0.003	0.068	positive	3040-38-8
Carnitine	up	1.31	0.011	0.154	positive	541-15-1
Coenzyme A (CoA)	up	2.82	0.028	0.282	positive	85-61-0
Pyruvate	up	1.43	0.028	0.238	negative	127-17-3
Acetoacetic Acid	up	1.40	0.028	0.238	negative	541-50-4
**Cholesterol Metabolites**						
Taurodeoxycholate	up	1.94	0.003	0.068	positive	516-50-7
Cholesteryl Acetate	up	2.24	0.008	0.192	negative	604-35-3
Lanosterol	up	2.50	0.011	0.192	negative	79-63-0
**Amino Acids**						
Tyrosine	up	1.25	0.037	0.270	negative	60-18-4
Serine	down	1.18	0.049	0.270	negative	56-45-1
Glutamate	down	1.08	0.049	0.282	positive	56-86-0
**Cysteine and Methionine Metabolites**						
Pterin	down	3.91	0.002	0.192	negative	2236-60-4
**Histidine Metabolites**						
Urocanic acid	down	1.34	0.021	0.212	negative	104-98-3
Carnosine	up	1.32	0.028	0.282	positive	305-84-0
**Arginine and Proline Metabolites**						
Phosphocreatine	up	1.62	0.037	0.282	positive	67-07-2
4-Guanidinobutyric Acid	down	1.09	0.049	0.270	negative	463-00-3
**Threonine Metabolites**						
2-Ketobutyric Acid	up	1.43	0.021	0.212	negative	600-18-0
**Tyrosine Metabolites**						
Homovanillic Acid	down	1.30	0.049	0.270	negative	306-08-1
**Riboflavin Metabolites**						
2, 4-Dihydroxypteridine (Lumazine)	up	2.07	0.008	0.154	positive	487-21-8
Flavin adenine dinucleotide (FAD)	up	1.19	0.021	0.212	negative	146-14-5
Lumichrome	down	1.71	0.049	0.282	positive	1086-80-2
**Pyrimidine Metabolites**						
2-Aminoisobutyric acid	up	1.38	0.011	0.154	positive	62-57-7
UMP	up	1.22	0.049	0.270	negative	58-97-9
CMP	up	1.29	0.049	0.282	positive	63-37-6
**Miscellaneous**						
Lipoic Acid	up	3.76	0.005	0.192	negative	1077-28-7
Thiourea	down	1.45	0.008	0.192	negative	62-56-6
Butyrylcarnitine	up	1.24	0.011	0.154	positive	25576-40-6
Hydroxybutyric Acid	up	1.49	0.011	0.192	negative	
Indoxyl Sulfate	down	2.12	0.021	0.212	negative	2642-37-7
3-Hydroxymethylglutaric Acid	up	1.27	0.028	0.238	negative	503-49-1
2-Hydroxypyridine	up	1.45	0.037	0.282	positive	142-08-5
Maleic acid	up	1.92	0.037	0.270	negative	110-16-7
N-Acetylneuraminic Acid	up	1.17	0.037	0.282	positive	131-48-6
N-Methylglutamic Acid	down	1.19	0.049	0.270	negative	35989-16-3
2-Aminoadipic Acid	down	1.17	0.049	0.270	negative	542-32-5
Cytidine diphosphate choline (CDPcholine)	down	1.17	0.049	0.282	positive	987-78-0
Glutathione	up	1.49	0.049	0.282	positive	70-18-8

We used PIUMet to analyze the 304 untargeted metabolite features whose levels were differentially altered in the EC of 14–15 month-old male *APOE4/4* vs. *APOE3/3* mice (corrected p-value < 0.05). Among these features, 124 had matches in PIUMet’s underlying database of metabolites (Supplementary Table S5). PIUMet was able to infer the identity of 32 metabolite features and revealed a network of protein-protein and protein-metabolite interactions associated with their dysregulation. The resulting network is enriched in 18 putative GO biological processes (Table 3) and 124 total metabolites (Supplementary Fig. S4). Among these, the most intriguing GO biological processes were fatty acid metabolic process, inosine 5′-monophosphate (IMP) metabolic process, ketone catabolic process, steroid metabolic process, and vitamin metabolic process, while the most interesting putative metabolite identifications were acetone, adenylosuccinate, alpha-tocotrienol, and oleamide. In regards to this study, the identification of acetone and the ketone catabolic process GO term are particularly interesting. Ketone bodies (acetone, acetoacetone and β-hydroxybutyrate) are primarily generated in the liver from fatty acid breakdown via β-oxidation, and are used as a supplemental energy source when glucose levels are low. These ketone bodies readily cross the blood brain barrier and can act as a potent fuel for the brain^35–37^. Alternatively, there is evidence that astrocytes also have the ability to produce ketones^38–40^, both through the breakdown of fatty acids, as well as through the breakdown of ketogenic amino acids such as leucine^41,42^. Thus, the putative identification of acetone and the ketone catabolic process GO term in this analysis may point to an upregulation of ketone metabolism in the EC of these aged *APOE4* mice.

**Table 3:** GO biological processes observed in the PIUMet analysis of differentially expressed untargeted metabolites from the EC of aged male *APOE4/4* vs. *APOE3/3* mice.

GO Term	Enrichment	p-value	FDR
carboxylic acid transport	12.35	9.80E-20	1.43E-16
carboxylic acid transmembrane transport	22.34	1.36E-16	1.04E-13
long-chain fatty acid metabolic process	18.08	2.77E-13	1.50E-10
fatty acid metabolic process	7.8	1.45E-11	5.40E-09
indolalkylamine metabolic process	44.68	2.48E-09	6.68E-07
disaccharide metabolic process	63.29	6.92E-09	1.77E-06
IMP metabolic process	48.69	3.47E-08	7.43E-06
steroid metabolic process	6.87	1.78E-07	3.24E-05
maltose metabolic process	126.58	4.81E-07	8.06E-05
kynurenine metabolic process	50.63	7.51E-07	1.18E-04
IMP biosynthetic process	50.63	7.51E-07	1.19E-04
tryptophan catabolic process	50.63	7.51E-07	1.20E-04
'de novo’ IMP biosynthetic process	63.29	9.46E-06	1.13E-03
ketone catabolic process		3.90E-05	3.92E-03
dopamine metabolic process	20.25	4.12E-05	4.06E-03
neurotransmitter catabolic process	37.97	5.54E-05	5.25E-03
keratan sulfate catabolic process	31.65	1.00E-04	8.37E-03
vitamin metabolic process	6.38	3.60E-04	2.35E-02

### Proton nuclear magnetic resonance spectroscopy analysis reveals differential expression of phosphocreatine, glutamate and GABA in the EC of anesthetized aged *APOE4* mice

**Figure 5:**
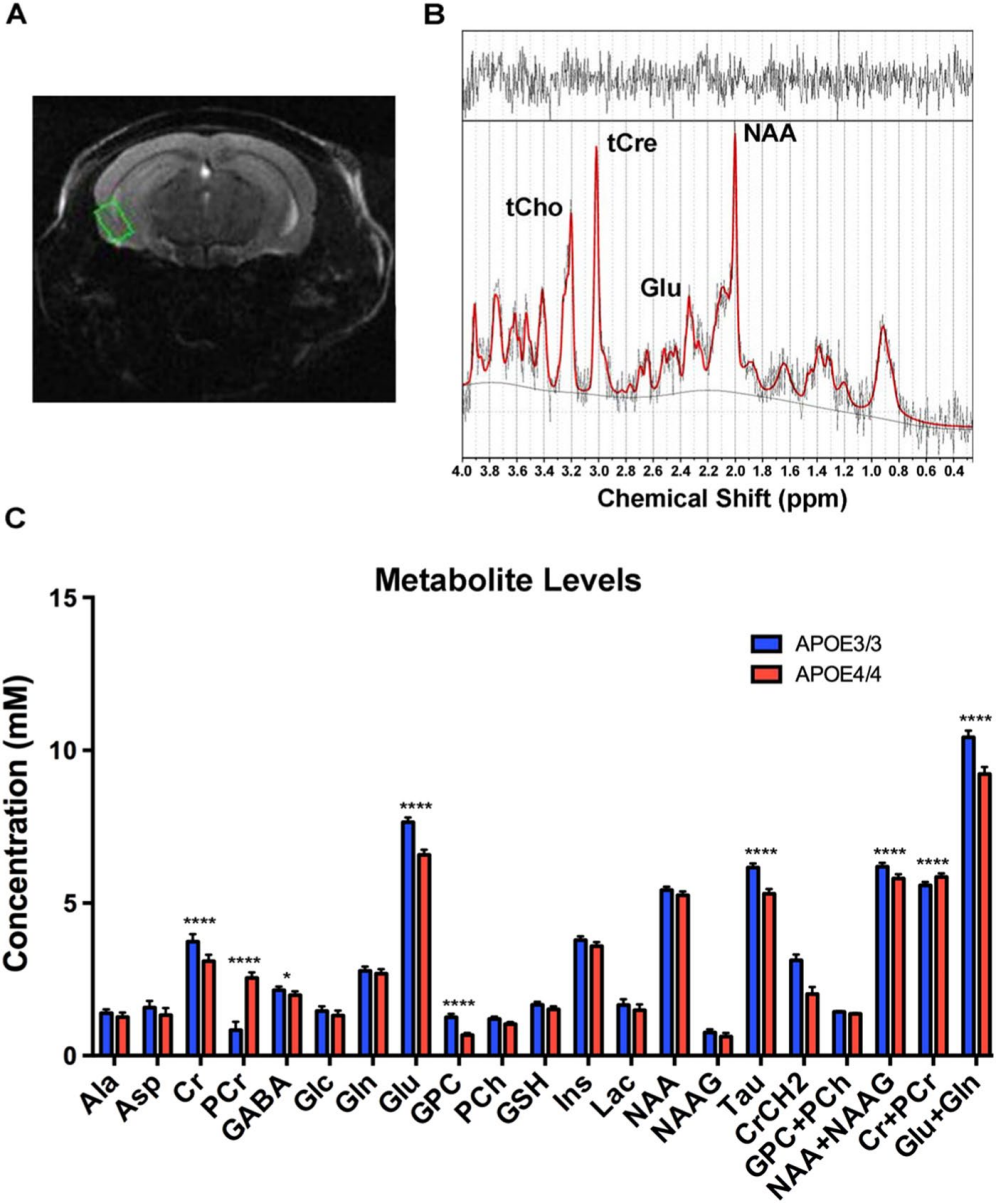
Proton nuclear magnetic resonance spectroscopy reveals differential expression of phosphocreatine, glutamate and GABA in the EC of aged male *APOE4* mice. ^1^H NMR spectroscopy was performed in order to analyze the effects of differential *APOE* isoform expression on the levels of high-abundance metabolites in the EC of anesthetized *APOE* mice (7 *APOE4/4* males vs. 7 *APOE3/3* males). (**A**) A representative coronal anatomical image used in the analysis, with the volume of interest, highlighted in green, shown over the EC. (**B**) A typical spectrum from the EC showing the spectral position of some major metabolites: N-Acetylaspartic acid (NAA), glutamate (Glu), total creatine (tCr) and total choline (tCho). The raw spectrum is shown in black (with no smoothing), and the software fit of the model spectra is shown in red. The top of the figure shows the difference between the raw spectrum and the fit, with a “good fit” showing random oscillations with little noise. The solid black line at the bottom of the figure is the baseline. (**C**) The metabolite concentrations in the EC of aged *APOE3/3* and *APOE4/4* mice. The metabolites reported are: alanine (Ala), aspartate (Asp), creatine (Cr), phosphocreatine (PCr), gamma-aminobutyric acid (GABA), glucose (Glc), glutamine (Gln), glutamate (Glu), glycerophosphocholine (GPC), phosphocholine (PCh), glutathione (GSH), myo-inositol (Ins), lactate (Lac), N-Acetylaspartate (NAA), N-Acetylaspartateglutamate (NAAG), taurine (Tau), total choline (GPC and PCh), total NAA (NAA and NAAG), total creatine (Cr and PCr) and total glutamate and glutamine (Glu and Gln). (* denotes p < 0.05; ****denotes p < 0.0001).

^1^H NMR spectroscopy provides simultaneous quantification of the concentration of several highly abundant metabolites in the brain, including neurotransmitters such as glutamate and gamma-aminobutyric acid (GABA), as well as other biological metabolites such as creatine, glutathione, and N-acetylaspartate (NAA). Since metabolomics and ^1^H NMR spectroscopy have been described as complementary techniques, with advantages and disadvantages to both [see review by^43^], we chose to perform ^1^H NMR spectroscopy on the EC of 18–19 month-old *APOE* mice (7 *APOE3/3* and 7 *APOE4/4* males) in order to gain additional information about the metabolic changes occurring in the EC of aged *APOE* mice. While the EC’s close proximity to sinus spaces makes the NMR analysis of this brain region challenging, we were able to achieve full width half maximums of 8–15 Hz using a customized second and third order shim coil. Figure 5A shows the position of the volume of interest (VOI) that was used to measure metabolite levels in the EC. A representative spectrum from this region is shown in Fig. 5B, along with the spectral position of some of the major metabolites. To our knowledge, this is the first *in vivo* ^1^H NMR study of the EC performed in mice.

As shown in Fig. 5C and Table 4, this ^1^H NMR study revealed significant differences in the levels of multiple metabolites in the EC of aged male *APOE4/4* vs. *APOE3/3* mice, including decreased levels of creatine (Cre), glutamate (Glu), GABA, and taurine (Tau). Perhaps the most interesting observation, however, was the large increases in the level of phosphocreatine (PCr) and the ratio of PCr:Cr observed in the *APOE4* EC. Creatine is mainly phosphorylated in mitochondria, where creatine kinase uses the newly synthesized ATP to produce PCr and ADP^44^. This PCr is then shuttled to the cytosol where it acts as a rapidly mobilized reserve of phosphate groups that can be used to replenish ATP levels, whereas the increased levels of ADP in the mitochondria will stimulate oxygen uptake and oxidative metabolism, especially during enhanced neuronal activity^45^. Thus, the observation that PCr levels are increased in aged *APOE4/4* mice supports and expands upon the bioenergetics observations from our transcriptomics, metabolomics and Seahorse analyses. In addition to these changes in PCr levels, the observation that glutamate and GABA are both decreased in the EC of *APOE4/4* mice may also be related to these bioenergetic differences between *APOE* genotypes and brain regions. Although glutamate and GABA are mostly known for their roles as neurotransmitters in excitatory and inhibitory neurons, respectively, they can also be utilized as energy metabolites^46–49^.

**Table 4:** Metabolite concentrations in the EC of aged male *APOE3/3* and *APOE4/4* mice, as observed by ^1^H NMR spectroscopy.

Metabolite	*APOE3/3* concentration (mM)	*APOE4/4* concentration (mM)	p-value
Creatine (Cr)	3.73 +/− 0.25	3.09 +/− 0.21	3.04E-04
Phosphocreatine (PCr)	0.84 +/− 0.27	2.55 +/− 0.18	1.00E-05
Gamma-aminobutyrate (GABA)	2.14 +/− 0.12	1.98 +/− 0.13	0.036
Glutamate (Glu)	7.65 +/− 0.15	6.58 +/− 0.17	5.30E-05
Glycerophosphocholine (GPC)	1.25 +/− 0.12	0.67 +/− 0.07	2.90E-05
Taurine (Tau)	6.16 +/− 0.14	5.31 +/− 0.15	1.59E-05
Cr + PCr	5.58 +/− 0.11	5.85 +/− 0.12	8.90E-04
Glu + Gln	10.43 +/− 0.21	9.22 +/− 0.23	2.30E-05
NAA + NAAG	6.19 +/− 0.14	5.81 +/− 0.12	8.90E-04

Taken together, our data suggest that, in contrast to other brain region such as the hippocampus and cortex, the EC of aged *APOE4* mice exhibits an increased rate of oxidative metabolism and ATP turnover, as compared to aged *APOE3* mice. We hypothesize that this differential regional mitochondrial respiration that we observe here is downstream of the decreased glucose utilization observed in *APOE4* carriers^16–18^ and may be related to the neuronal hyperactivity that we observed in the EC of aged *APOE4* mice^33^. In addition, we hypothesize that the unique bioenergetic compensatory mechanisms that we observe in the EC may lead to an increased rate of reactive oxygen species (ROS) generation in this region, which may play a causative role in the pathogenesis of AD among *APOE4* carriers.

## Discussion

Possession of the *APOE4* allele greatly increases an individual’s risk of developing AD. Although numerous theories have been proposed, the precise cause of this association remains unknown. In order to further investigate the effects of *APOE4* gene expression in AD-relevant brain regions, we initially chose to perform a transcriptomics analysis on RNA extracted from an AD-vulnerable brain region (the EC) vs. a less vulnerable brain region (the PVC) of 14–15 month-old male *APOE* targeted replacement mice. In addition to our previously published report describing *APOE4*′s impact on endosomal-lysosomal dysregulation^30^, this analysis also revealed *APOE4*-associated alterations in the levels of numerous genes related to energy metabolism (Fig. 1). Follow-up experiments utilizing the Seahorse platform revealed *APOE4*-associated deficits in mitochondrial respiration with age in several brain regions, including the PVC, the Hip, and the Ctx as a whole (Fig. 3, Supplemental Fig. S1 and Supplementary Fig. S3). This observation is consistent with previous reports showing that *APOE4* expression has numerous deleterious effects on the brain’s bioenergetic capabilities^19–24^, although it is important to note that the decline in mitochondrial functionality that we observed in the cortex and hippocampus, while significant, is likely below the pathological threshold^50^. Intriguingly, however, this *APOE4*-associated decrease in mitochondrial respiration with age was not observed in the EC. Additional studies, including proton nuclear magnetic resonance (^1^H NMR) spectroscopy and a bioinformatics analysis of our untargeted metabolomics data provide further support and elucidation of this region-specific bioenergetic regulation by *APOE4*, including the observations of increased PCr levels and decreased glutamate and GABA levels (Fig. 5), as well as an upregulation of the ketone pathway (Table 3), in the EC of aged *APOE4* mice.

Mitochondria are part of a multifaceted network of metabolic pathways that are modulated by the cell to compensate for any reductions in respiratory activity and ATP production^51,52^. Mitochondria have been described as having a “reserve respiratory capacity” that allows them to increase their oxidative capacity and ATP production depending on the cell’s energy demands^53,54^. Furthermore, mitochondrial adaptability is tissue and cell-type specific, depending on the different compensatory mechanisms and the availability of substrates^50,55^. Perhaps the most intriguing finding from our study, however, is that, in addition to these tissue and cell-type specific differences in mitochondrial adaptability, it appears that within the brain, mitochondria also have *regional* differences in their response to metabolic stressors such as *APOE4* expression.

As shown in previous studies, the brains of *APOE4* carriers display decreased glucose utilization in numerous AD-relevant brain regions, as observed by FDG-PET^16–18^. We hypothesize that the differential regional bioenergetic characteristics observed in the current study may represent region-specific responses to the decreased glucose utilization caused by *APOE4* expression. For example, in both the EC and the Ctx as a whole, it appears that there are attempts to use alternative energy sources, such as the β-oxidation of fatty acids, to compensate for *APOE4*′s effects on glucose utilization. This is represented by the decreased fatty acid levels and increased carnitine levels observed in the metabolomics data from both the EC and PVC of the aged male *APOE4/4* vs. *APOE3/3* mice. In addition, the increased Complex II-mediated mitochondrial respiration observed in both the EC and the Ctx of aged female *APOE4/4* vs. *APOE3/3* mice suggests that aged female *APOE4* mice may utilize β-oxidation of fatty acids to an even greater extent than the aged male *APOE4* mice. However, the data from our Seahorse and ^1^H NMR spectroscopy studies, as well as further mining of our transcriptomics and metabolomics data, suggests that the mitochondria in the EC of aged *APOE4* mice also utilize additional mechanisms to counteract any *APOE4*-associated reductions in glucose utilization, with the net effect being an increased coupling efficiency between oxygen consumption and ATP production.

This additional response in the EC is most clearly observed in our Seahorse data, where mitochondria in the EC of aged male *APOE4* mice show increased ADP-stimulated oxygen consumption and increased maximal respiration rate, without changes in proton leak (Fig. 3) or mitochondrial mass (Supplementary Fig. S2). In addition, we also observed that the RCR is increased in the EC of aged male *APOE4/4* vs. *APOE3/3* mice (Fig. 3C), further confirming that ADP-driven mitochondrial respiration in the aged *APOE4* EC is better coupled to ATP production than in the Hip, the PVC and the Ctx. Interestingly, the reduction in mitochondrial respiration observed in the Ctx of both aged male and female *APOE4/4* vs. *APOE3/3* mice by Seahorse analysis appears to be more robust in Complex I-mediated respiration compared to Complex II-mediated respiration (Fig. 3 and Supplementary Fig. S3), whereas there is no decreased respiration in the EC of aged female *APOE4/4* mice (Supplementary Fig. S3), and in the EC of aged male *APOE4/4* mice, this Complex I-mediated respiration actually begins to outpace the Complex I-mediated respiration observed in the aged male *APOE3/3* mice (Fig. 3). Since Complex I-mediated mitochondrial respiration is driven by pyruvate, this suggests that the *APOE4*-associated bioenergetic deficits observed in the Hip and Ctx of aged *APOE4* mice are primarily related to decreased aerobic respiration that occurs downstream of glycolysis, whereas this decreased aerobic respiration does not appear to occur in the EC of these mice.

While the reason for the increased mitochondrial coupling in the EC of aged *APOE4* mice requires further investigation, we note that our transcriptomics data reveals an increased expression of some mitochondrial transporter genes in this brain region (Supplementary Tables S1 and S2). Mitochondrial transporters link biochemical pathways in the cytosol and mitochondria in order to ensure a sufficient rate of solute flux into mitochondria to fuel metabolic pathways^56^. Regulation of these transporters is an essential part of the overall regulation of cellular metabolism, and their expression is highly variable depending on the tissues and the metabolic conditions. Intriguingly, the expression of the gene for adenine nucleotide translocase 1 (ANT1; *Slc25a4*) was significantly increased in the EC of aged male *APOE3/4* vs. *APOE3/3* mice. ANT1 participates in ATP-for-ADP exchange through the inner mitochondrial membrane, which supplies the cytoplasm with newly synthesized ATP from OxPhos. It can also interact with creatine kinase (CK), gaining preferential access to ATP, which is needed to synthesize PCr in cells with high energetic needs^57^. Similarly, expression of the gene for the voltage dependent-anion channel 1 (VDAC1; Vdac1), one of the main gatekeepers for the regulation of the crosstalk between mitochondria and cytosol^56^, is also increased in the EC of these aged *APOE3/4* mice, as is the gene for the mitochondrial phosphate transport protein (PTP; *Slc25a3*), which transports inorganic phosphate (P_i_) to the mitochondrial matrix in order to supply the P_i_ required for ADP phosphorylation into ATP^56^. Another important mitochondrial transporter system that ensures the balanced cooperation between OxPhos and glycolysis (and to a lesser extent the pentose phosphate pathway) in order to maintain ATP levels is the malate-aspartate shuttle. In order to transfer the two NADH molecules generated during glycolysis into mitochondria, the enzymes malate dehydrogenase (MDH1) and glutamate oxaloacetate transaminase 1 (GOT1) mediate a series of metabolic conversions involving malate, oxaloacetate, aspartate and glutamate. Interestingly, our transcriptomics data reveals that the expression of both Slc25a22 (GOT1) and *Mdh1* (MDH1) is upregulated in the EC of aged male *APOE3/4* vs. *APOE3/3*, suggesting that the activity of the malate-aspartate shuttle is increased in the EC of these mice.

**Figure 6:**
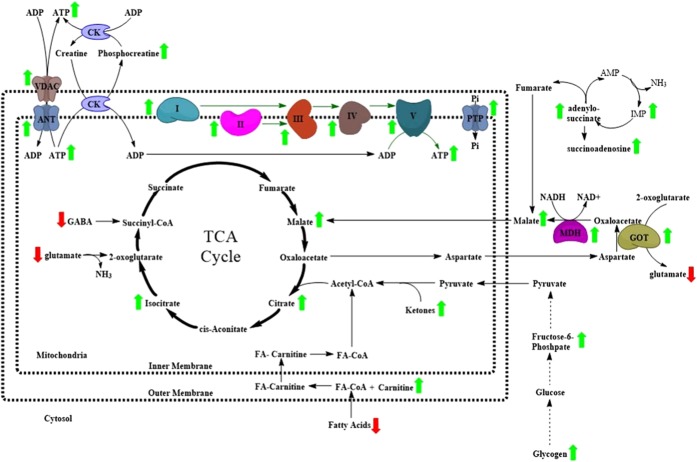
Identification of differentially expressed bioenergetic genes and metabolites in the EC of aged *APOE4* mice. A schematic of the bioenergetics genes and metabolites that have been predicted to be altered by differential *APOE* isoform expression in this study, as depicted in a simplified manner with respect to their relationships to each other and their location inside or outside of mitochondria. Genes or metabolites that were putatively identified as increased in aged male *APOE4* mice, as compared to aged male *APOE3* mice, are marked with a green arrow, and those that are decreased are marked with a red arrow.

A summary of all these bioenergetics-related changes that we observe in the EC of aged male *APOE4* mice is depicted in Fig. 6. Taken together, the observed differences on mitochondrial functionality among the brain regions studied suggest that the EC possesses a different subset of compensatory mechanisms in order to improve OxPhos efficiency under metabolic stress. While this apparent metabolic compensation in the EC may be regarded as a positive attribute, it may have negative consequences in the long run. For example, it has been shown that over time, elevated mitochondrial respiratory activity and increased coupling efficiency result in an excess of free radical production and resulting oxidative damage within cells^58,59^. In fact, mild mitochondrial uncoupling, and the resulting increase in proton leak and decrease in mitochondrial membrane potential, has been previously suggested as a neuroprotective mechanism that cells utilize to prevent ROS formation by blocking the accumulation of electrons at early steps of the transport chain^60^.

The fact that we observe increased Complex II-mediated mitochondrial respiration in the Ctx and EC of aged female *APOE4* mice (Supplementary Fig. S3) adds further complexity to this story, but it also adds further evidence of increased ROS production in the brains of these aged *APOE4* mice. When glucose supply is not high enough to provide a cell with sufficient ATP to meet its energy demands, the cell may resort to the ß-oxidation of fatty acids in order to help fuel the electron transport chain. During this ß-oxidation process, these fatty acids are metabolized into acetyl-CoA molecules, which feed into the TCA cycle, a system that includes the conversion of succinate to fumarate by Complex II. However, unlike glucose metabolism, which generates a high ratio of NADH:FADH_2_ (about 5:1), the ß-oxidation of fatty acids generates a much lower NADH:FADH_2_ ratio (about 2:1 for palmitate, for example)^61^. This increased FADH_2_ ratio can increase the production of ROS, both by maintaining the electron transport chain in a highly reduced state due to limited NADH supply, as well as by increasing the activity of flavoprotein-ubiquinone oxidoreductase, which is a potent source of superoxide production^61,62^. Thus, although the EC from the aged female *APOE4* mice did not require as much compensatory activity in its Complex I-mediated mitochondrial respiration as what we observed in the aged male *APOE4* mice, it is possible that the presence of a compensatory effect in Complex I-mediated respiration in the EC of aged female *APOE4* mice, in combination with an overall increase in ROS production throughout the brain of these mice resulting from the increased Complex II-mediated respiration, may lead to even greater pathogenic effects in the EC of aged female vs. aged male *APOE4* mice.

More work will of course be necessary in order to further elucidate this phenomenon, including an understanding of the specific contribution of different cell-types, as well as determining whether these same EC-specific compensatory mechanisms that we observed in the aged *APOE4* mice exist in the EC of human *APOE4* carriers. Given the limitations of human imaging studies and the lack of published omics data on human EC tissues from *APOE4* vs. *APOE3* carriers, it is difficult to directly compare our results to published human data at the present moment. It has been reported, however, that AD patients who possess at least one *APOE4* allele show increased relative tau accumulation^63,64^ and increased brain atrophy^65,66^ in the EC compared to *APOE4* non-carriers. Importantly, there is also evidence of EC-specific changes in human *APOE4* carriers long before any symptoms of AD appear. For example, decreased entorhinal thickness has been observed in both young and middle-aged *APOE4* carriers as compared to non-carriers^67,68^, and young adult *APOE4* carriers exhibit reduced EC-related grid-cell-like representations than non-carriers^69^. Furthermore, middle-aged *APOE4* carriers have been shown to possess increased incorporation of docosahexaenoic acid (DHA) specifically in the EC region compared to non-carriers^70^, a finding that may be related to results from our metabolomics study, where DHA was the only fatty-acid observed to be upregulated in the EC of aged *APOE4/4* vs. *APOE3/3* mice (Fig. 2B). Although the reason for this *APOE4*-associated upregulation of DHA incorporation in the EC is unknown, it is interesting to note that DHA concentrations have been found to be positively correlated to the rates of energy metabolism in cells and tissues^71^.

It will also be important to investigate whether the other phenotypes that we have observed in the EC of these aged *APOE4* mice, such as endosomal-lysosomal dysregulation and neuronal hyperactivity, are related to the bioenergetic observations described here. For example, Zhao *et al*. reported that *APOE4* expression causes insulin receptors to be inefficiently recycled via the endosomal-lysosomal system, potentially causing the insulin resistance that the authors observed in the brains of aged *APOE4* mice^20^. As the endosomal-lysosomal dysregulation that we observed in the brains of aged *APOE4* mice was not restricted to the EC^30^, this may play a role in the *APOE4*-associated bioenergetic deficits that we observed in the Hip and Ctx of these mice. On the other hand, the neuronal hyperactivity that we observed appears to be primarily localized to the EC within the hippocampal formation^33^. Intriguingly, neuronal activity and glucose utilization have previously been shown to exist in a near 1:1 stoichiometric relationship^72^, suggesting that the bioenergetic counterbalancing that we observe in the EC of aged *APOE4* mice may be directly correlated to the observed neuronal hyperactivity in these mice^33^.

Finally, it will be important to determine whether these bioenergetic differences in the EC are involved in the increased risk of AD among *APOE4* carriers. The EC is a unique brain region in many ways. It is vital for learning and memory (especially spatial memory), acting as a bridge between the hippocampus and the neocortex^73,74^. As such, it is known to have higher energy demands and to be more metabolically active that many other brain regions^75^. During AD pathogenesis, the EC is one of the first regions to develop tauopathy, which eventually develops in the hippocampus and other cortical regions as the disease progresses^76^. It is possible, therefore, that due to its unique bioenergetic needs, the EC may possess compensatory mechanisms for increasing its bioenergetic capacity under conditions of metabolic stress such as *APOE4* expression. Alternatively, these compensatory mechanisms may be initiated in order to fuel the greater energy demands that come from neuronal hyperactivity in the EC prior to AD onset. Regardless of the cause, it is possible that, in the process of inducing these compensatory bioenergetic mechanisms, other negative consequences may result, such as increased ROS production and increased tau hyperphosphorylation/aggregation, such that it results in an accelerated pathogenesis of AD.

## Methods

### Mice

Human APOE targeted replacement mice were first developed by Sullivan *et al*.^28,29^ and were acquired from Dr. Sullivan, or from Taconic Biosciences. All mice used in this study were treated in accordance with the National Institutes of Health Guide for the Care and Use of Laboratory Animals and approved by the Columbia University Medical Center and the Nathan Kline Institute’s Institutional Animal Care and Use Committee (IACUC).

### RNA-sequencing

Transcriptomics analysis of aged *APOE* mice was performed as previously described^30^. Briefly, male mice expressing human *APOE3/3* (10 mice) or *APOE3/4* (19 mice) were aged to 14–15 months, at which point they were sacrificed by cervical dislocation, and brain tissues containing the EC and PVC were dissected and snap frozen on dry-ice. Brain tissues were stored in RNase-free eppendorf tubes at −80 °C prior to extraction. Total RNA was extracted from frozen tissues by homogenizing each tissue sample using a battery-operated pestle mixer (Argos Technologies, Vernon Hills, IL) in 1 ml of TRIzol reagent according to the manufacturer’s protocol (Life Technologies, Carlsbad, CA). RNA concentration was measured using a nanodrop 1000 (Thermo Fisher Scientific, Waltham, MA), and RNA integrity (RIN) was assessed using an Agilent 2100 Bioanalyzer (Agilent Technologies, Santa Clara, CA). All RNA samples possessed a RIN of 8 or higher. RNA was stored at −80 °C prior to use. Starting with 2 μg of total RNA per sample, Poly(A) + mRNA was purified, fragmented and then converted into cDNA using the TruSeq RNA Sample Prep Kit v2 (Illumina cat# RS-122–2001) as per the manufacturer’s protocol (Illumina, San Diego, CA). For RNA-Sequencing of the cDNA, we hybridized 5 pM of each library to a flow cell, with a single lane for each sample, and we used an Illumina cluster station for cluster generation. We then generated 149 bp single end sequences using an Illumina HiSeq 2000 sequencer. For analysis, we used the standard Illumina pipeline with default options to analyze the images and extract base calls in order to generate fastq files. We then aligned the fastq files to the mm9 mouse reference genome using Tophat (v2.0.6) and Bowtie (v2.0.2.0). In order to annotate and quantify the reads to specific genes, we used the Python module HT-SEQ with a modified NCBIM37.61 (containing only protein coding genes) gtf to provide reference boundaries. We used the R/Bioconductor package DESeq 2 (v1.10.1) for comparison of aligned reads across the samples. We conducted a variance stabilizing transformation on the aligned and aggregated counts, and then the Poisson distributions of normalized counts for each transcript were compared across *APOE3/4* vs. *APOE3/3* groups using a negative binomial test. We corrected for multiple testing using the Benjamini-Hochberg procedure and selected all genes that possessed a corrected p-value of less than 0.05. Finally, a heat map based on sample distance and a volcano plot based on fold change and adjusted p-values were generated using the R/Bioconductor package ggplot2 (v2.0.0). For pathway analysis, enriched KEGG pathways were identified using the ClueGo application (version 2.1.7) in Cytoscape (version 3.2.1). Briefly, all differentially expressed EC genes from the RNA-Seq analysis were entered into the application and searched for significantly enriched KEGG pathways possessing a Benjamini-Hochberg adjusted p-value of less than 0.05.

### Seahorse analysis

In order to investigate the effect of differential *APOE* isoform expression on electron transport chain activity, mitochondria were isolated from tissues collected from 15-month-old male mice expressing *APOE3/3* (4 mice) or *APOE4/4* (4 mice) (experiment 1), from 6-month-old male mice expressing *APOE3/3* (3 mice) or *APOE4/4* (3 mice) (experiment 2), from 20-month-old male mice expressing *APOE3/3* (2 mice) and *APOE4/4* (2 mice) (experiment 3), and from 20-month-old female mice expressing *APOE3/3* (2 mice) and *APOE4/4* (2 mice) (experiment 4). Tissue samples were pooled by region and genotype, and the oxygen consumption rates (OCR) were measured in a Complex I or Complex II-mediated fashion, as previously described^77^. Briefly, each mouse was sacrificed by cervical dislocation, and the different brain areas from each brain hemisphere were immediately removed and placed into ice-cold PBS containing protease inhibitors. Following tissue collection, samples were homogenized in ~10 volumes of isolation buffer (70 mM sucrose, 210 mM mannitol, 5 mM HEPES, 1 mM EGTA and 0.5% fatty acid-free BSA, pH 7.2) and rinsed 3 times. Tissues were then homogenized using a Teflon glass homogenizer with 2–3 strokes. Homogenized samples were then centrifuged at 800 × g for 10 min at 4 °C. Following centrifugation, fat/lipid was aspirated off, and the remaining supernatant was decanted through two layers of cheesecloth into a separate tube and centrifuged at 8000 × g for 10 min at 4 °C. After removal of the supernatant, the pellet was resuspended in mitochondrial isolation buffer, and the centrifugation was repeated. The resulting pellet was resuspended in mitochondrial isolation buffer, and total protein concentrations were determined using Bradford Assay reagent (Bio-Rad). For Complex I experiments, 8 μg of protein was added to each well and for Complex II analysis 6 μg per well. To prepare samples for OCR measurements, samples were prepared in mitochondrial assay buffer (70 mM sucrose, 220 mM mannitol, 10 mM KH2PO4, 5 mM MgCl2, 2 mM HEPES, 1 mM EGTA and 0.2% fatty acid-free BSA, pH 7.2) plus substrate (pyruvate and malate for the Complex I-mediated ETC activity assay, or succinate and rotenone for the Complex II-mediated ETC activity assay).

Respirometry was performed using the XF24e Extracellular Flux Analyzer (Seahorse Bioscience). Oxygen consumption was measured in basal conditions (Seahorse media with 25 mM glucose and 2 mM pyruvate) and after the sequential addition of 1 μM oligomycin (Complex V inhibitor), 0.75 μM FCCP (uncoupler) and 1 μM rotenone/1 μM antimycin A (Complex I and Complex III inhibitors, respectively). All results are averages of five or more biological replicates. Every biological replicate consisted of three technical replicates. OCR data was generated by the Seahorse XF24 1.5.0.69 software package and displayed in point-to-point mode. Final calculations of total OCR were performed by normalizing *APOE4/4* values to *APOE3/3* for each of the three experiments and then combining the data in Prism.

### Metabolomic analysis

Metabolomics analysis of aged *APOE* mice was performed as previously described^33^. Briefly, male mice expressing human *APOE3/3* (8 mice), *APOE3/4* (9 mice), or *APOE4/4* (7 mice) were aged to 14–15 months, at which point they were sacrificed by cervical dislocation to maintain the brain environment, and individual brain regions were immediately removed and snap-frozen on dry ice. Tissues were stored at −80 °C for prior to extraction. Metabolite extraction was performed using a methyl tert-butyl ether (MTBE)/methanol extraction protocol modified from previous reports^78,79^. Briefly, individual EC or PVC tissues were homogenized in 400 μl of ice-cold methanol using a bead mill homogenizer (TissueLyser II, Qiagen) at 25 beats/sec, 2x for 45 sec each. Following homogenization, samples were incubated in 1200 μl of MTBE for 1 hr at room temperature to separate organic-soluble lipids from aqueous-soluble lipids and other small molecules. Finally, 360 μl of ultrapure water was added (for a final ratio of 3:1:0.9 MTBE:methanol:water) to resolve the two liquid phases, and each sample was centrifuged at 10,000 × g for 10 min. The lower aqueous phase and the upper organic phase were collected from each sample and stored in separate tubes, and the remaining protein pellets were resuspended in 25 mM ammonium bicarbonate, pH 8, with 2.5% SDS. A BCA protein assay was performed on each protein fraction, and both the aqueous phase and organic phase were normalized to their protein concentration equivalent with 50% and 100% methanol, respectively. All samples were then stored at −80 °C prior to analysis. Metabolite profiling was performed using an Agilent Model 1200 liquid chromatography (LC) system coupled to an Agilent Model 6230 time-of-flight (TOF) mass analyzer as described previously^80^. Metabolite separation was accomplished using aqueous neutral phase (ANP) gradient chromatography on a Diamond Hydride column (Microsolv), with mobile phase buffer A consisting of 50% isopropanol and 0.025% acetic acid, and mobile phase buffer B consisting of 90% acetonitrile and 5 mM ammonium acetate. Each aqueous phase sample was analyzed in both positive and negative ion detection mode for comprehensive coverage of metabolites with a mass of 50–1000 Da. Prior to analysis, it was observed that one *APOE3/4* PVC sample possessed a chromatographic error and was removed from all future analyses.

Following MS analysis, the raw data was analyzed using Agilent Profinder (version B.06.00) and Mass Profiler Professional (version 12.0) software. Briefly, Profinder performs batch recursive feature extraction, which consists of a first-pass identification of features (ions) that possess a common elution profile (e.g. identical mass-to-charge [m/z] ratios and retention times), followed by a recursive analysis to re-mine each sample for the presence of features that were identified in at least one sample. Feature extraction was limited to the top 2000 metabolites per ion detection mode, with each metabolite possessing a minimum abundance of 600 ion counts. Each feature was also manually validated following extraction (with investigators blinded to genotype assignments during validation), and re-integration was performed when necessary. In addition to this untargeted feature extraction, we also used Profinder to perform a targeted feature extraction, matching features against a proprietary database of 626 biologically-relevant metabolites whose mass and retention times were previously determined using pure chemical standards. For this analysis, we allowed for a maximum mass deviation of 10 mDa and a retention time deviation of 1 min, and identified features were manually validated following extraction. For both untargeted and targeted feature extraction, Profinder analysis was followed up by multivariate and differential expression analysis using Mass Profiler Professional. Following differential expression analysis, metabolites identified as differentially expressed were further validated for chromatographic and biological accuracy, and non-validated metabolites were removed.

### PIUMet analysis

We used the previously published PIUMet algorithm^34^ to analyze the untargeted metabolomics data from the EC of aged *APOE4/4* vs. *APOE3/3* mice. PIUMet discovers dysregulated molecular pathways and components associated with changes in untargeted metabolomics data by analyzing a network built from curated protein-protein and protein-metabolite interactions (PPMI). PIUMet represents each metabolite peak as a node in this network and connects it to metabolites with masses that correspond to the m/z values of the peak. Using the prize-collecting Steiner tree algorithm, it searches the PPMI interactome to find an optimum subnetwork that connects the input metabolite peaks via their putative identities and other metabolites and proteins that were not detected in the experiments. The Gene Ontology (GO) analysis of the resulting network further reveals molecular pathways associated with the *APOE4* genotype. We selected 304 untargeted metabolite peaks or features that were significantly altered between the aged *APOE4/4* and *APOE3/3* mice (corrected p-value < 0.05). We then assigned a prize to each input data point to show the significance of their alterations, as –log (P values) of the significance of changes between two phenotypic conditions. PIUMet accepts several parameters that regulate the size of the resulting networks, including w and beta. While w tunes the number of trees in the resulting network, beta tunes the number of input nodes that are included in the output. Another parameter, mu, controls the bias toward high degree nodes. Higher values of mu results in a lower number of high degree nodes in the resulting networks, and thus less bias to highly studied molecules or ubiquitous interactions such as those with ions. We determined the optimum parameters (w = 6.0, beta = 0.5, and mu = 0.0005) based on their effects on the size of the resulting networks. We further generated 100 networks by adding random noise to the underlying database, and calculated robustness scores for each node. Nodes with robustness scores less than 60% were removed from the results. Additionally, for each node we calculated a specificity score, based on the number of their presence in the 100 random networks obtained from a set of mock metabolite peaks randomly selected from the input feature list that mimic real input. A score of 100 indicates that the node did not show in any of the randomly generated networks, while a zero score shows the node appears in all the randomly generated networks. 79% of the resulting nodes have specificity scores of over 60%.

### ^1^H NMR spectroscopy

All animal procedures for *in vivo* proton MR spectroscopy were performed as described previously^81,82^, and in accordance with the National Institutes of Health guidelines with approval from the Institutional Animal Care and Use Committee at the Nathan S. Kline Institute for Psychiatric Research. All animals were anesthetized using an isoflurane vaporizer set at the following percentages: 3% for induction, 2% during pilot scanning and 1.5% during data acquisition. An animal monitoring unit (model 1025, SA Instruments, Stony Brook, NY, USA) was used to record respiration and rectal temperature. Respiration was measured with a pressure transducer placed under the abdomen just below the ribcage. Body temperature was maintained using forced warm air, controlled by a feedback circuit between the heater and thermistor. After induction, the animals were placed on a holder and restrained using a bite bar and ear bars placed half way into the auditory canal. Oxygen was used as the carrier gas delivered to a cone positioned before the bite bar, where gases mixed with air and passed over the rodent’s nose. All animals were maintained at 37.0 ± 0.2 °C. All data were obtained on a 7.0T Agilent (Santa Clara, CA, USA) 40 cm bore system. The gradient coil insert had an internal diameter of 12 cm with a maximum gradient strength of 600 mT/m and minimum rise time of 200 μs, with customized second and third order shim coils. A Rapid (Rimpar, Germany) volume transmit coil (72 mm ID) and a two-channel receive-only surface coil was used for RF transmission and reception, respectively.

The shim settings for the selected volume of interest (VOI) were automatically adjusted using FASTMAP^1^ (Fast, Automatic Shimming Technique by Mapping Along Projections), a high order shim method, which samples the magnetic field along a group of radial columns which focus on the center of a localized voxel. It is a method for optimizing the field homogeneity in a cubical local region. The water signal was suppressed using variable power RF pulses with optimized relaxation delays (VAPOR)^2^. The spectral acquisition consisted of a short echo time Point Resolved Spectroscopy (PRESS)^3^ sequence with the following parameters: repetition time = 4 s, echo time = 7.5 ms, number of averages = 512 acquired in blocks of 128, number of points = 2048 and bandwidth of acquisition = 5 kHz, total acquisition time = 34 minutes. Outer volume suppression was also used to minimize signal contamination by extra cranial muscle and lipids. The VOI size was 3.3 µl (2.0 × 1.3 × 1.3 mm^3^) placed in the EC. The target VOI is depicted in Fig. 5 overlaid on a schematic of the coronal brain slice at approximately – 2.92 mm relative to Bregma. An anatomical T_2_ weighted pilot scan was used to position the VOI (coronal). These scans were acquired with a fast spin echo sequence with the following parameters: field of view = 20 mm with 256 × 256 matrix size, slice thickness = 0.5 mm, number of slices = 11, repetition time = 4 s, echo train length = 8, echo spacing = 15 ms, effective echo time = 60 ms, number of averages = 8, total acquisition time = 8 minutes 40 s. All data were processed using the LCModel software developed by Provencher^4^. This software calculates the best fit to the acquired data of a linear combination of model spectra acquired from *in vitro* solutions of all the brain metabolites of interest. This basis set of the model spectra has the same echo time, sequence acquisition and field strength as the acquired data of the study. The LCModel software outputs the estimated concentration along with estimated standard deviations (Cramer-Rao lower bounds) expressed in percent of the estimated concentration, which can be used as a quantitative measure of reliability. To improve statistical significance, Miller *et al*. recently demonstrated the use of weighted averaging in NMR spectroscopic studies^83^. In this current study, we also used weighted averaging to calculate the standard unequal variance t-test (Welch’s t-test) as outlined by Miller^83^ and in the reference manual of the LCModel software^84^. All data used in the final analysis had Cramer-Rao lower bounds of 20% or less.

The metabolites measured were: alanine (Ala), aspartate (Asp), creatine (Cr), phosphocreatine (PCr), ɣ-Aminobutyric Acid (GABA), glucose (Glc), glutamine (Gln), glutamate (Glu), glycerophosphocholine (GPC), phosphocholine (PCh), glutathione (GSH), myo-inositol (Ins), lactate (Lac), N-Acetylaspartate (NAA), N-Acetylaspartateglutamate (NAAG) and taurine (Tau). It is often quite difficult to resolve Glu from Gln, NAA from NAAG and PCh from GPC, particularly if the spectral quality is poor due to an inadequate shim. So, in addition to the principal metabolites, the total of Glu and Gln, NAA and NAAG, and PCh and GPC are also reported. These total concentrations are thought to be a more reliable metric. An unsuppressed water signal was also used for absolute concentration calculation and eddy current correction. This internal reference method assumes known values of water concentrations of gray and white matter^5–7^.

There are several limitations to ^1^H NMR spectroscopy which can make reliable measures particularly challenging. One such limitation is that is that ^1^H NMR metabolite measures are inherently low in signal to noise. The metabolite signals measured are approximately 10,000-fold less than the proton signal used in imaging. The EC is also a small structure and thus requires a small VOI which reduces the measured signal strength. However, at 7T with shim values in the range 8–15 Hz and a short echo time of 8 ms, the spectral peaks were reasonably separated, allowing for direct and reliable measures of metabolite concentrations. For metabolites which have significant spectral overlap, total values (e.g. Glu and Gln) are also reported. Another error source is chemical shift displacement. The bandwidth of the 180° RF pulse is 5 kHz, which would give a displacement error of 0.3 mm in the vertical and horizontal directions over the range of 0.2 to 4.2 ppm. However, this displacement was relatively small and still allowed for sufficient specificity. Despite the challenges of *in vivo* mouse brain ^1^H NMR, we were able to achieve high quality spectra in this difficult region with high quantification accuracy.

## Supplementary information

Supplementary Information.

Supplementary Figure 4 - high resolution.

## Data Availability

The transcriptomics datasets are available in the NCBI Gene Expression Omnibus (GEO) repository: https://www.ncbi.nlm.nih.gov/geo/query/acc.cgi?acc=GSE102334. The metabolomics datasets are available in the MetaboLights repository: http://www.ebi.ac.uk/metabolights/MTBLS530.

## References

[1] Mahley RW, Rall SC (2000). Apolipoprotein E: far more than a lipid transport protein. Annu. Rev. Genomics Hum. Genet..

[2] Han X (2004). The role of apolipoprotein E in lipid metabolism in the central nervous system. Cell Mol. Life Sci..

[3] Holtzman DM, Herz J, Bu G (2012). Apolipoprotein E and apolipoprotein E receptors: normal biology and roles in Alzheimer disease. Cold Spring Harb. Perspect. Med..

[4] Liu CC, Kanekiyo T, Xu H, Bu G (2013). Apolipoprotein E and Alzheimer disease: risk, mechanisms and therapy. Nat. Rev. Neurol..

[5] Farrer LA (1997). Effects of age, sex, and ethnicity on the association between apolipoprotein E genotype and Alzheimer disease. A meta-analysis. APOE and Alzheimer Disease Meta Analysis Consortium. JAMA.

[6] Rubinsztein DC, Easton DF (1999). Apolipoprotein E genetic variation and Alzheimer’s disease. a meta-analysis. Dement. Geriatr. Cogn. Disord..

[7] Bales KR (1997). Lack of apolipoprotein E dramatically reduces amyloid beta-peptide deposition. Nat. Genet..

[8] Castano EM (1995). Fibrillogenesis in Alzheimer’s disease of amyloid beta peptides and apolipoprotein E. Biochem. J..

[9] Rebeck GW, Reiter JS, Strickland DK, Hyman BT (1993). Apolipoprotein E in sporadic Alzheimer’s disease: allelic variation and receptor interactions. Neuron.

[10] Schmechel DE (1993). Increased amyloid beta-peptide deposition in cerebral cortex as a consequence of apolipoprotein E genotype in late-onset Alzheimer disease. Proc. Natl Acad. Sci. U S Am..

[11] Ma J, Yee A, Brewer HB, Das S, Potter H (1994). Amyloid-associated proteins alpha 1-antichymotrypsin and apolipoprotein E promote assembly of Alzheimer beta-protein into filaments. Nat..

[12] Castellano JM (2011). Human apoE isoforms differentially regulate brain amyloid-beta peptide clearance. Sci. Transl. Med..

[13] Holtzman DM (2000). Apolipoprotein E isoform-dependent amyloid deposition and neuritic degeneration in a mouse model of Alzheimer’s disease. Proc. Natl Acad. Sci. U S Am..

[14] Huang Y (2010). Abeta-independent roles of apolipoprotein E4 in the pathogenesis of Alzheimer’s disease. Trends Mol. Med..

[15] Wolf AB (2013). Apolipoprotein E as a beta-amyloid-independent factor in alzheimer’s disease. Alzheimers Res. Ther..

[16] Reiman EM (1996). Preclinical evidence of Alzheimer’s disease in persons homozygous for the epsilon 4 allele for apolipoprotein E. N. Engl. J. Med..

[17] Reiman EM (2004). Functional brain abnormalities in young adults at genetic risk for late-onset Alzheimer’s dementia. Proc. Natl Acad. Sci. U S Am..

[18] Reiman EM (2005). Correlations between apolipoprotein E epsilon4 gene dose and brain-imaging measurements of regional hypometabolism. Proc. Natl Acad. Sci. U S Am..

[19] Ong QR, Chan ES, Lim ML, Cole GM, Wong BS (2014). Reduced phosphorylation of brain insulin receptor substrate and Akt proteins in apolipoprotein-E4 targeted replacement mice. Sci. Rep..

[20] Zhao N (2017). Apolipoprotein E4 Impairs Neuronal Insulin Signaling by Trapping Insulin Receptor in the Endosomes. Neuron.

[21] Johnson, L. A. *et al*. Apolipoprotein E4 mediates insulin resistance-associated cerebrovascular dysfunction and the post-prandial response. *Journal of cerebral blood flow and metabolism: official journal of the International Society of Cerebral Blood Flow and Metabolism*, 271678X17746186 (2017). 10.1177/0271678X17746186 PMC6498752 29215310

[22] Johnson LA, Torres ER, Impey S, Stevens JF, Raber J (2017). Apolipoprotein E4 and Insulin Resistance Interact to Impair Cognition and Alter the Epigenome and Metabolome. Sci. Rep..

[23] Keeney JT, Ibrahimi S, Zhao L (2015). Human ApoE Isoforms Differentially Modulate Glucose and Amyloid Metabolic Pathways in Female. Brain: Evid. Mechanism Neuroprotection ApoE2 Implic. Alzheimer’s Dis. Prev. Early Intervention. J. Alzheimer’s disease: JAD..

[24] Wu L, Zhang X, Zhao L (2018). Human ApoE Isoforms Differentially Modulate Brain Glucose and Ketone Body Metabolism: Implications for Alzheimer’s Disease Risk Reduction and Early Intervention. J. neuroscience: Off. J. Soc. Neurosci..

[25] Chang S (2005). Lipid- and receptor-binding regions of apolipoprotein E4 fragments act in concert to cause mitochondrial dysfunction and neurotoxicity. Proc. Natl Acad. Sci. U S Am..

[26] Huang Y (2001). Apolipoprotein E fragments present in Alzheimer’s disease brains induce neurofibrillary tangle-like intracellular inclusions in neurons. Proc. Natl Acad. Sci. U S Am..

[27] Nakamura T, Watanabe A, Fujino T, Hosono T, Michikawa M (2009). Apolipoprotein E4 (1-272) fragment is associated with mitochondrial proteins and affects mitochondrial function in neuronal cells. Mol. Neurodegener..

[28] Sullivan PM, Mace BE, Maeda N, Schmechel DE (2004). Marked regional differences of brain human apolipoprotein E expression in targeted replacement mice. Neurosci..

[29] Sullivan PM (1997). Targeted replacement of the mouse apolipoprotein E gene with the common human APOE3 allele enhances diet-induced hypercholesterolemia and atherosclerosis. J. Biol. Chem..

[30] Nuriel T (2017). The Endosomal-Lysosomal Pathway Is Dysregulated by APOE4 Expression *in Vivo*. Front. Neurosci..

[31] Rogers GW (2011). High throughput microplate respiratory measurements using minimal quantities of isolated mitochondria. PLoS One.

[32] Brand MD, Nicholls DG (2011). Assessing mitochondrial dysfunction in cells. Biochem. J..

[33] Nuriel T (2017). Neuronal hyperactivity due to loss of inhibitory tone in APOE4 mice lacking Alzheimer’s disease-like pathology. Nat. Commun..

[34] Pirhaji L (2016). Revealing disease-associated pathways by network integration of untargeted metabolomics. Nat. methods.

[35] Robinson AM, Williamson DH (1980). Physiological roles of ketone bodies as substrates and signals in mammalian tissues. Physiological Rev..

[36] Zhang Y (2013). Ketosis proportionately spares glucose utilization in brain. J. Cereb. blood flow. metabolism: Off. J. Int. Soc. Cereb. Blood Flow. Metab..

[37] Lin AL, Zhang W, Gao X, Watts L (2015). Caloric restriction increases ketone bodies metabolism and preserves blood flow in aging brain. Neurobiol. aging.

[38] Thevenet J (2016). Medium-chain fatty acids inhibit mitochondrial metabolism in astrocytes promoting astrocyte-neuron lactate and ketone body shuttle systems. FASEB journal: Off. Publ. Federation Am. Societies Exp. Biol..

[39] Auestad N, Korsak RA, Morrow JW, Edmond J (1991). Fatty acid oxidation and ketogenesis by astrocytes in primary culture. J. neurochemistry.

[40] Guzman M, Blazquez C (2004). Ketone body synthesis in the brain: possible neuroprotective effects. Prostaglandins, leukotrienes, Essent. Fat. acids.

[41] Bixel MG, Hamprecht B (1995). Generation of ketone bodies from leucine by cultured astroglial cells. J. neurochemistry.

[42] Murin R, Hamprecht B (2008). Metabolic and regulatory roles of leucine in neural cells. Neurochem. Res..

[43] Emwas AH (2015). The strengths and weaknesses of NMR spectroscopy and mass spectrometry with particular focus on metabolomics research. Methods Mol. Biol..

[44] Bessman SP (1987). The creatine phosphate energy shuttle–the molecular asymmetry of a “pool”. Anal. Biochem..

[45] Chen W, Zhu XH, Adriany G, Ugurbil K (1997). Increase of creatine kinase activity in the visual cortex of human brain during visual stimulation: a 31P magnetization transfer study. Magnetic Reson. Med..

[46] Schousboe A, Bak LK, Waagepetersen HS (2013). Astrocytic Control of Biosynthesis and Turnover of the Neurotransmitters Glutamate and GABA. Front. Endocrinol..

[47] Kreft, M., Bak, L. K., Waagepetersen, H. S. & Schousboe, A. Aspects of astrocyte energy metabolism, amino acid neurotransmitter homoeostasis and metabolic compartmentation. *ASN neuro* **4** (2012). 10.1042/AN20120007 PMC3338196 22435484

[48] Falkowska A (2015). Energy Metabolism of the Brain, Including the Cooperation between Astrocytes and Neurons, Especially in the Context of Glycogen Metabolism. Int. J. Mol. Sci..

[49] Patel AB (2005). The contribution of GABA to glutamate/glutamine cycling and energy metabolism in the rat cortex *in vivo*. Proc. Natl Acad. Sci. USA.

[50] Rossignol R, Malgat M, Mazat JP, Letellier T (1999). Threshold effect and tissue specificity. Implic. mitochondrial cytopathies. J. Biol. Chem..

[51] Celotto AM, Chiu WK, Van Voorhies W, Palladino MJ (2011). Modes of metabolic compensation during mitochondrial disease using the Drosophila model of ATP6 dysfunction. PLoS One.

[52] Chan DC (2006). Mitochondria: dynamic organelles in disease, aging, and development. Cell.

[53] Sansbury BE, Jones SP, Riggs DW, Darley-Usmar VM, Hill BG (2011). Bioenergetic function in cardiovascular cells: the importance of the reserve capacity and its biological regulation. Chemico-biological Interact..

[54] Pfleger J, He M, Abdellatif M (2015). Mitochondrial complex II is a source of the reserve respiratory capacity that is regulated by metabolic sensors and promotes cell survival. Cell Death Dis..

[55] Goodpaster BH, Sparks LM (2017). Metabolic Flexibility in Health and Disease. Cell Metab..

[56] Palmieri F (2013). The mitochondrial transporter family SLC25: identification, properties and physiopathology. Mol. Asp. Med..

[57] Saks VA (1995). Control of cellular respiration *in vivo* by mitochondrial outer membrane and by creatine kinase. A new speculative hypothesis: possible involvement of mitochondrial-cytoskeleton interactions. J. Mol. Cell. cardiology.

[58] Bratic I, Trifunovic A (2010). Mitochondrial energy metabolism and ageing. Biochimica et. biophysica acta.

[59] Vesce S, Kirk L, Nicholls DG (2004). Relationships between superoxide levels and delayed calcium deregulation in cultured cerebellar granule cells exposed continuously to glutamate. J. neurochemistry.

[60] Korshunov SS, Skulachev VP, Starkov AA (1997). High protonic potential actuates a mechanism of production of reactive oxygen species in mitochondria. FEBS Lett..

[61] Schonfeld P, Reiser G (2013). Why does brain metabolism not favor burning of fatty acids to provide energy? Reflections on disadvantages of the use of free fatty acids as fuel for brain. J. Cereb. Blood Flow. Metab..

[62] Quinlan CL (2012). Mitochondrial complex II can generate reactive oxygen species at high rates in both the forward and reverse reactions. J. Biol. Chem..

[63] Whitwell JL (2018). [(18) F]AV-1451 clustering of entorhinal and cortical uptake in Alzheimer’s disease. Ann. Neurol..

[64] Mattsson N (2018). Greater tau load and reduced cortical thickness in APOE epsilon4-negative Alzheimer’s disease: a cohort study. Alzheimers Res. Ther..

[65] Geroldi C (1999). APOE-epsilon4 is associated with less frontal and more medial temporal lobe atrophy in AD. Neurol..

[66] Juottonen K, Lehtovirta M, Helisalmi S, Riekkinen PJ, Soininen H (1998). Major decrease in the volume of the entorhinal cortex in patients with Alzheimer’s disease carrying the apolipoprotein E epsilon4 allele. J. neurology, neurosurgery, psychiatry.

[67] Shaw P (2007). Cortical morphology in children and adolescents with different apolipoprotein E gene polymorphisms: an observational study. Lancet Neurol..

[68] Burggren AC (2008). Reduced cortical thickness in hippocampal subregions among cognitively normal apolipoprotein E e4 carriers. Neuroimage.

[69] Kunz L (2015). Reduced grid-cell-like representations in adults at genetic risk for Alzheimer’s disease. Sci..

[70] Yassine HN (2017). DHA brain uptake and APOE4 status: a PET study with [1-(11)C]-DHA. Alzheimers Res. Ther..

[71] Brenna JT, Diau GY (2007). The influence of dietary docosahexaenoic acid and arachidonic acid on central nervous system polyunsaturated fatty acid composition. Prostaglandins Leukot. Essent. Fat. Acids.

[72] Yellen G (2018). Fueling thought: Management of glycolysis and oxidative phosphorylation in neuronal metabolism. J. Cell Biol..

[73] Eichenbaum H (2000). A cortical-hippocampal system for declarative memory. Nat. reviews. Neurosci..

[74] Frank LM, Brown EN, Wilson M (2000). Trajectory encoding in the hippocampus and entorhinal cortex. Neuron.

[75] Egorov AV, Hamam BN, Fransen E, Hasselmo ME, Alonso AA (2002). Graded persistent activity in entorhinal cortex neurons. Nat..

[76] Braak H, Braak E (1991). Neuropathological stageing of Alzheimer-related changes. Acta Neuropathol..

[77] Pera M (2017). Increased localization of APP-C99 in mitochondria-associated ER membranes causes mitochondrial dysfunction in Alzheimer disease. EMBO J..

[78] Matyash V, Liebisch G, Kurzchalia TV, Shevchenko A, Schwudke D (2008). Lipid extraction by methyl-tert-butyl ether for high-throughput lipidomics. J. Lipid Res..

[79] Giavalisco P (2011). Elemental formula annotation of polar and lipophilic metabolites using (13) C, (15) N and (34) S isotope labelling, in combination with high-resolution mass spectrometry. Plant. J..

[80] Chen Q (2012). Untargeted plasma metabolite profiling reveals the broad systemic consequences of xanthine oxidoreductase inactivation in mice. PLoS One.

[81] Baslow MH (2016). Stimulation-induced transient changes in neuronal activity, blood flow and N-acetylaspartate content in rat prefrontal cortex: a chemogenetic fMRS-BOLD study. NMR Biomed..

[82] Kaur G (2014). Glutamatergic transmission aberration: a major cause of behavioral deficits in a murine model of Down’s syndrome. J. neuroscience: Off. J. Soc. Neurosci..

[83] Miller JJ, Cochlin L, Clarke K, Tyler DJ (2017). Weighted averaging in spectroscopic studies improves statistical power. Magnetic Reson. Med..

[84] Provencher SW (1993). Estimation of metabolite concentrations from localized *in vivo* proton NMR spectra. Magnetic Reson. Med..

