# Supplementary material for: APOE4 is Associated with Differential Regional Vulnerability to Bioenergetic Deficits in Aged APOE Mice
Source: Sci Rep. 2020 Mar 9;10:4277. doi: 10.1038/s41598-020-61142-8 (PMC7062695; doi:10.1038/s41598-020-61142-8)

APOE4 is Associated with Differential Regional Vulnerability to Bioenergetic Deficits in Aged *APOE* Mice

Estela Area-Gomez[1,2], Delfina Larrea[1], Marta Pera[1,3], Rishi R. Agrawal[2], David N. Guilfoyle[4], Leila Pirhaji[5], Kathleen Shannon[6], Hirra A. Arain[7,8], Archana Ashok[78], Qiuying Chen[9], Allissa A. Dillman[10], Helen Y. Figueroa[7,8], Mark R. Cookson[10], Steven S. Gross[9], Ernest Fraenkel[5,11], Karen E. Duff[1,8,12], and Tal Nuriel[7,8,*]

* Corresponding author: Tal Nuriel, email: tn2283@cumc.columbia.edu

FIGURE LEGENDS

Supplementary Fig. S1. Seahorse analysis reveals decreased mitochondrial respiration in the PVC, but not in the EC, of aged male *APOE4* mice. Seahorse analysis was performed in order to analyze the effects of differential *APOE* isoform expression on mitochondrial respiration in mitochondria that were isolated from the PVC and EC of aged *APOE* mice (4 *APOE4/4* males, tissues pooled vs. 4 *APOE3/3* males, tissues pooled). (A) The complex I- and complex II-driven ETC activity from each region shows decreased mitochondrial respiration in the PVC, but not the EC of the aged *APOE4/4* mice. (B-C) Bar graphs showing the average oxygen consumption rate (OCR) from (B) State 3 and for (C) the Respiration Control Ratio (RCR; state 3u/state 4o) in each region of the *APOE4/4* mice, as a percentage of the *APOE3/3* OCR from the equivalent tissues. The dotted blue line represents the normalized levels in the *APOE3/3* tissues. (** denotes $p < 0.01$; *** denotes $p < 0.001$; **** denotes $p < 0.0001$)

Supplementary Fig. S2. Western blot and qPCR analysis shows no significant effects of APOE4 on mitochondrial mass or ETC protein expression. Western blotting and qPCR analysis was performed in order to investigate the effects of differential APOE isoform expression on various markers of mitochondrial mass and ETC proteins in the EC, Hip and Ctx of 21-month-old *APOE4/4* vs. *APOE3/3* mice (3 *APOE3/3* and 3 *APOE4/4* males). (A-B) Western blot analysis did not reveal any unique differences in the levels of mitochondrial outer membrane protein Tom20 or ETC complex subunits between genotypes in the EC. For each of these Western blot experiments, the samples were all run on the same gel, and then the blot was cut lengthwise into two separate segments in order to stain with the antibody of interest and the loading control, respectively. For the ETC complex staining, several exposure times were captured for the complex and then the clearest exposure time for each ETC subunit was utilized. We also did not observe any changes in (C) the ratio of Mitochondrial:Nuclear DNA, or (D) the levels of PGC1α RNA between *APOE* genotypes in the EC of 21-month-old *APOE* mice. (* denotes $p < 0.05$; ** denotes $p < 0.01$)

Supplementary Fig. S3. Seahorse analysis reveals decreased complex I-mediated mitochondrial respiration in the cortex, but not the EC of aged female *APOE4* mice, while complex II-mediated mitochondrial respiration is increased in both regions. Seahorse analysis was performed in order to analyze the effects of differential *APOE* isoform expression on mitochondrial respiration in mitochondria that were isolated from the cortex (Ctx), hippocampus (Hip) and EC of 20-month-old female *APOE* mice (2 *APOE4/4* females, tissues pooled vs. 2 *APOE3/3* females, tissues pooled). (A) The oxygen consumption rate (OCR) from the EC shows minor reductions in complex I-mediated mitochondrial respiration in the Ctx, but not in the Hip or EC. On the other hand, complex II-mediated mitochondrial respiration is increased in both the Ctx and the EC, but not the Hip. (B-C) Bar graphs showing the average oxygen consumption rate (OCR) from (B) State 3 and for (C) the Respiration Control Ratio (RCR; state 3u/state 4o) in each region of the *APOE4/4* mice, as a percentage of the *APOE3/3* OCR from the equivalent tissues. The dotted blue line represents the normalized levels in the *APOE3/3* tissues. (* denotes $p < 0.05$; ** denotes $p < 0.01$; *** denotes $p < 0.001$; **** denotes $p < 0.0001$)

Supplementary Fig. S4. Results network from the PIUMet analysis of differentially expressed untargeted metabolites from the EC of aged male *APOE4/4* vs. *APOE3/3* mice. Shown here is the results-network that was generated from the PIUMet (Prize-collecting Steiner forest algorithm for Integrative Analysis of Untargeted Metabolomics) analysis. PIUMet was able to predict identities for the differential expressed unidentified metabolite features or peaks detected in the EC of 14-15 month-old *APOE4/4 vs. APOE3/3* mice (corrected p-value <0.05, 8 *APOE3/3* and 7 *APOE4/4* males). The resulting network shows 242 nodes connected by 328 edges. Input metabolite peaks or features are shown as red triangles, while grey circles represent proteins and square nodes display the metabolites in the network. The metabolites directly connected to the differential metabolite features show their putative identities. The size of the nodes reflects the specificity score and the thickness of the edges the confidence of the interaction. In addition, the robustness score of each node is associated with the thickness of each node line width.

Supplementary Fig. S1

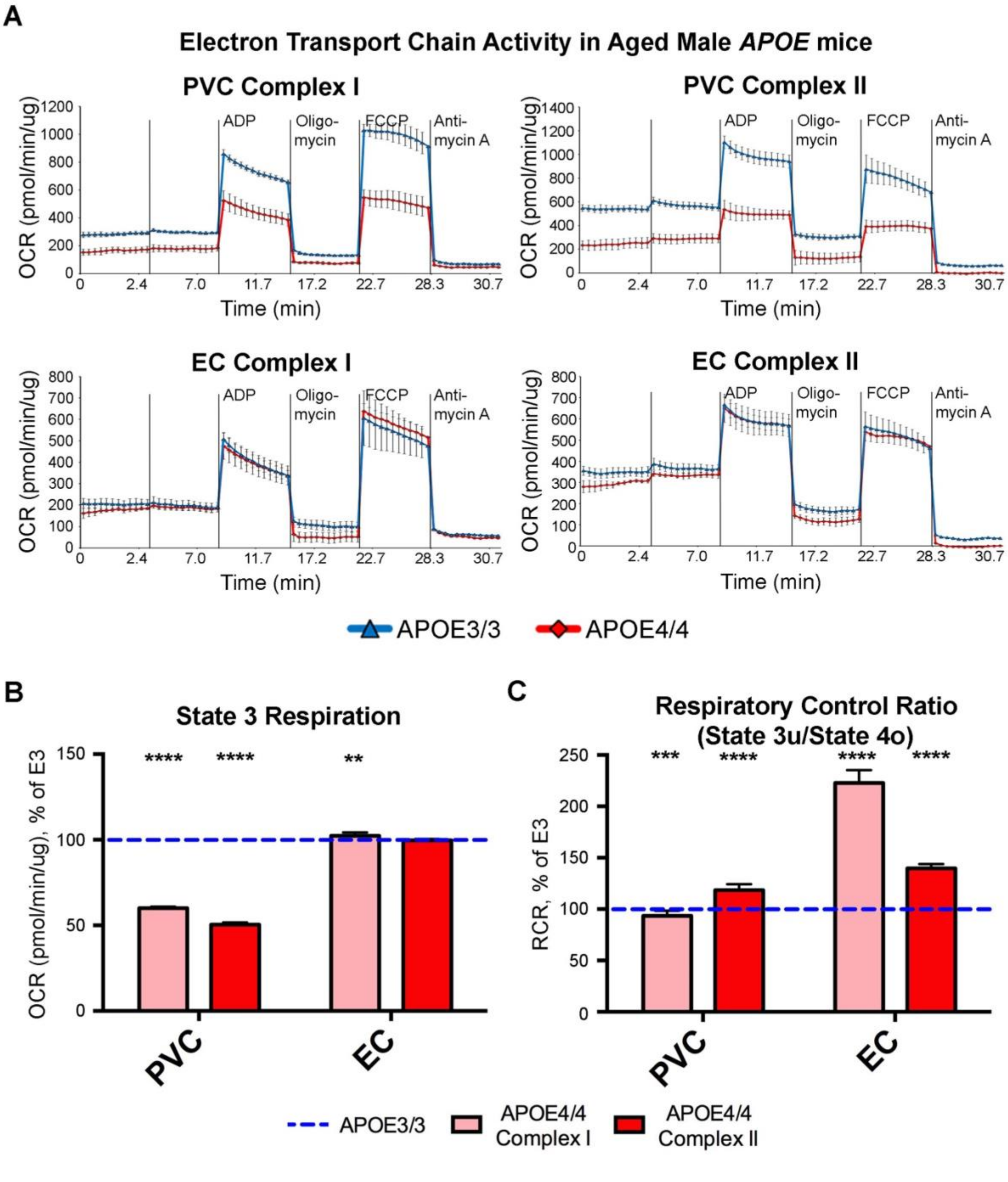

Supplementary Fig. S2

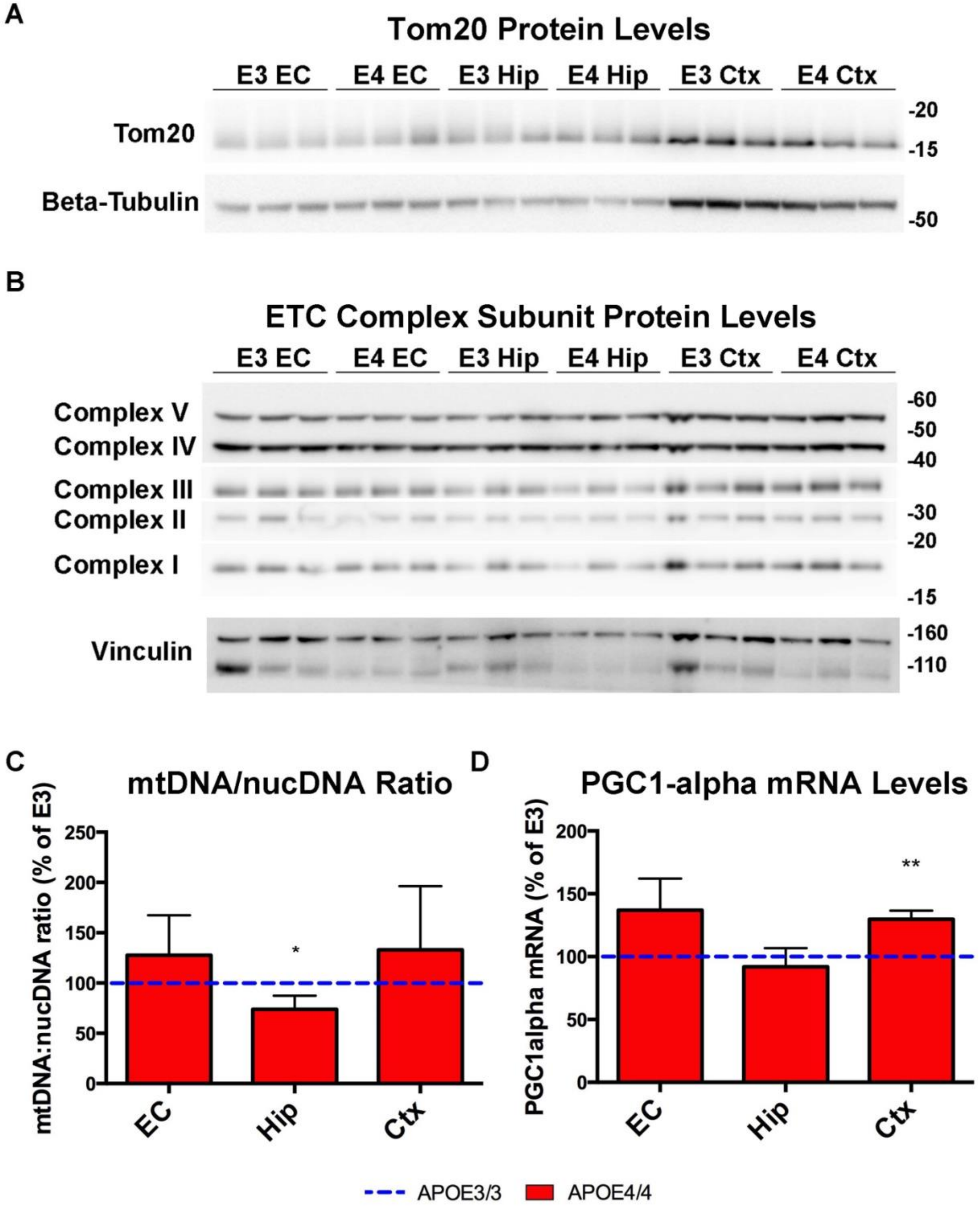

Supplementary Fig. S3

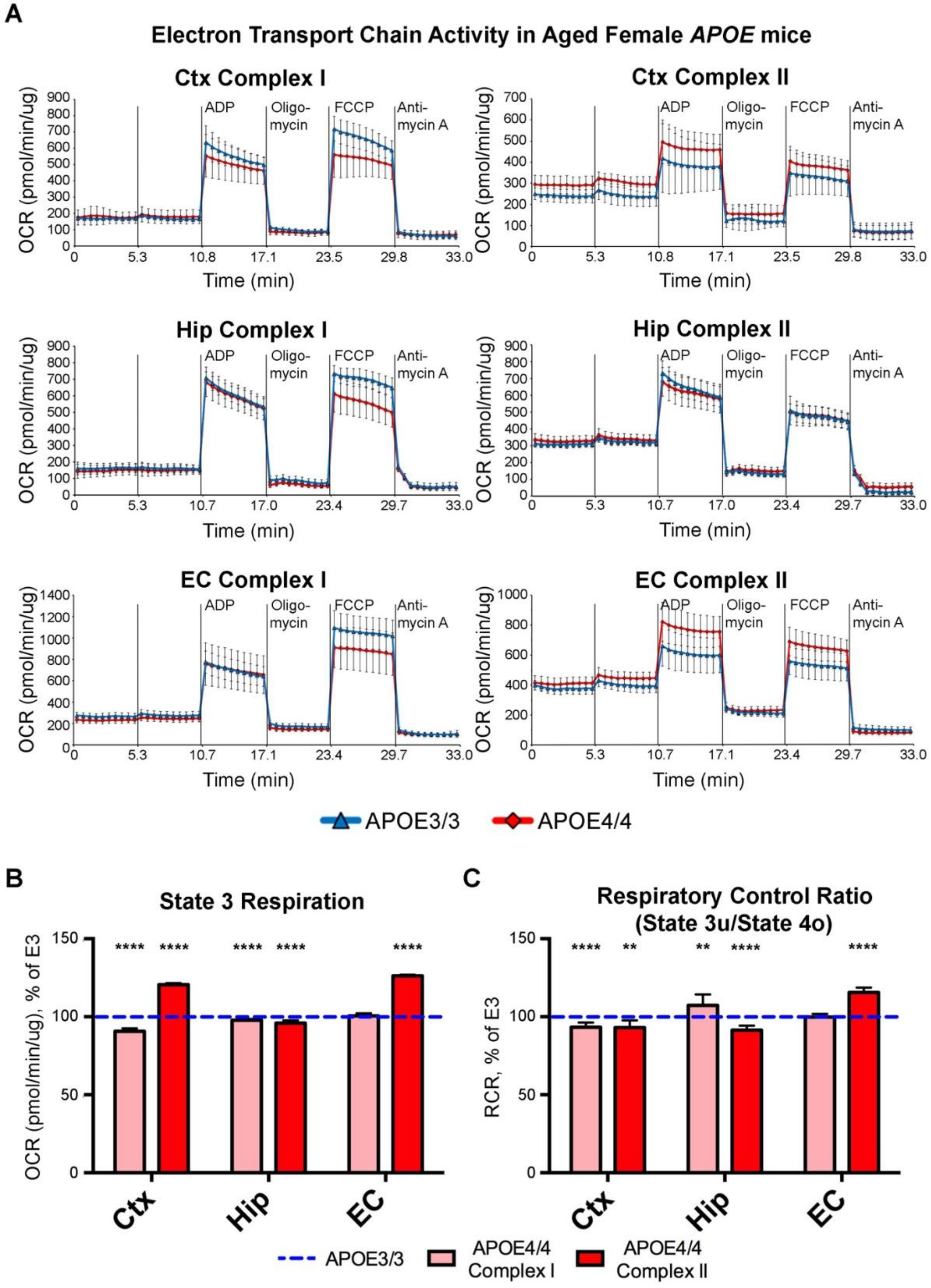

Supplementary Fig. S4 (high-res version available as supplemental file)

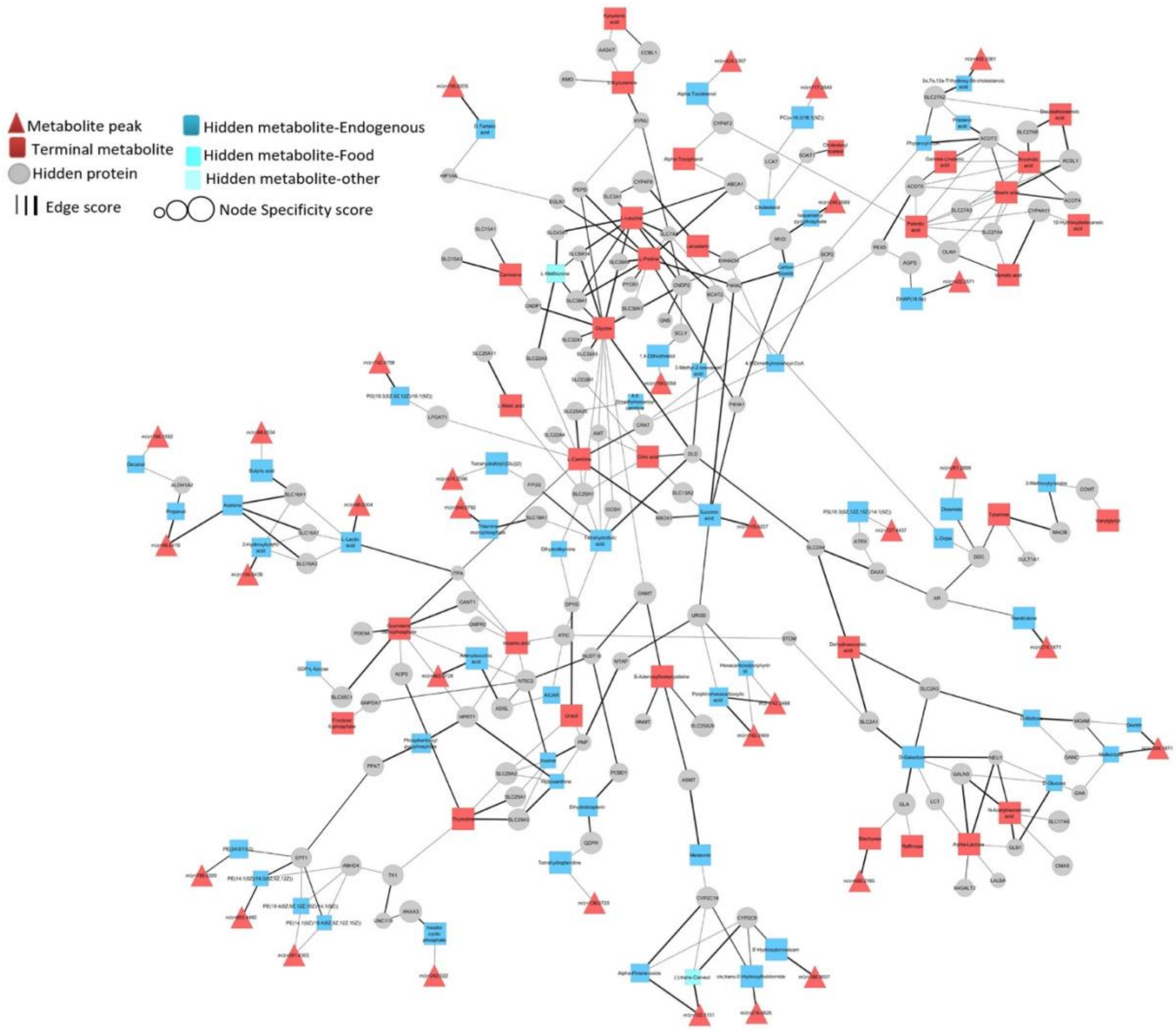

Supplement: Supplementary file 1 — Supplementary Information. [file 41598_2020_61142_MOESM1_ESM.pdf]
